# Age-Dependent Cell Trafficking Defects in Draining Lymph Nodes Impair Adaptive Immunity and Control of West Nile Virus Infection

**DOI:** 10.1371/journal.ppat.1005027

**Published:** 2015-07-23

**Authors:** Justin M. Richner, Grzegorz B. Gmyrek, Jennifer Govero, Yizheng Tu, Gerritje J. W. van der Windt, Talibah U. Metcalf, Elias K. Haddad, Johannes Textor, Mark J. Miller, Michael S. Diamond

**Affiliations:** 1 Department of Medicine, Washington University School of Medicine, St. Louis, Missouri, United States of America; 2 Department of Pathology & Immunology, Washington University School of Medicine, St. Louis, Missouri, United States of America; 3 Vaccine and Gene Therapy Institute of Florida, Port St. Lucie, Florida, United States of America; 4 Department of Theoretical Biology & Bioinformatics, Utrecht University, Utrecht, Netherlands; 5 Department of Molecular Microbiology, Washington University School of Medicine, St. Louis, Missouri, United States of America; Fox Chase Cancer Center, UNITED STATES

## Abstract

Impaired immune responses in the elderly lead to reduced vaccine efficacy and increased susceptibility to viral infections. Although several groups have documented age-dependent defects in adaptive immune priming, the deficits that occur prior to antigen encounter remain largely unexplored. Herein, we identify novel mechanisms for compromised adaptive immunity that occurs with aging in the context of infection with West Nile virus (WNV), an encephalitic flavivirus that preferentially causes disease in the elderly. An impaired IgM and IgG response and enhanced vulnerability to WNV infection during aging was linked to delayed germinal center formation in the draining lymph node (DLN). Adoptive transfer studies and two-photon intravital microscopy revealed a decreased trafficking capacity of donor naïve CD4^+^ T cells from old mice, which manifested as impaired T cell diapedesis at high endothelial venules and reduced cell motility within DLN prior to antigen encounter. Furthermore, leukocyte accumulation in the DLN within the first few days of WNV infection or antigen-adjuvant administration was diminished more generally in old mice and associated with a second aging-related defect in local cytokine and chemokine production. Thus, age-dependent cell-intrinsic and environmental defects in the DLN result in delayed immune cell recruitment and antigen recognition. These deficits compromise priming of early adaptive immune responses and likely contribute to the susceptibility of old animals to acute WNV infection.

## Author Summary

While West Nile virus (WNV) infection preferentially causes severe neuroinvasive disease in elderly humans, the basis for this epidemiological linkage has remained uncertain. Here, we studied the impact of aging on WNV pathogenesis and immune responses using a mouse model of infection. Old mice showed increased lethality after WNV infection compared to adult mice, and this phenotype was associated with delayed antibody responses, and higher levels of virus infection in the blood, spleen, and brain. Detailed immunological and microscopic analyses revealed defects in germinal center development in the draining lymph node and impaired migratory capacity of naïve CD4^+^ T cells within days of WNV infection. This deficit was worsened by a separate age-related deficiency in the production of several chemokines that recruit leukocytes to inflamed lymph nodes. Thus, age-dependent defects in naïve CD4^+^ T cell trafficking and in the draining lymph node environment result in delayed initiation of antiviral responses and a failure to control WNV, which ultimately contributes to higher rates of mortality after infection.

## Introduction

Aging is linked to a decline in immunity that causes increased susceptibility to infectious diseases and reduced vaccine efficacy in the elderly population. This process, termed immunosenescence, is the consequence of age-dependent changes to multiple components of the innate and adaptive immune responses. For example, elderly individuals display elevated basal levels of pro-inflammatory cytokines and type I interferon (IFN) [1], and demonstrate reduced signaling following pathogen-associated molecular pattern (PAMP) recognition [2]; collectively this results in dysregulated cytokine responses [3]. Additionally, elderly individuals exhibit a shift from a naïve to a memory repertoire in CD4^+^ T cells, CD8^+^ T cells [4–6], and B cells [7], which decreases the number of cells capable of recognizing new antigens encountered during primary infections. Cell-intrinsic defects also have been identified in different arms of the adaptive response. Naïve CD4^+^ T cells from old individuals display reduced activation, immunological synapse formation, and proliferation upon T cell receptor (TCR) engagement [8,9]. This leads to a diminished ability of CD4^+^ T cells to prime B cells during germinal center reactions [10,11] and, along with intrinsic B cell defects [12], results in reduced affinity maturation, class switching, and memory responses [11].

West Nile Virus (WNV) is a globally important mosquito-transmitted RNA virus that causes severe disease preferentially in the elderly as a consequence of enhanced dissemination to the central nervous system (CNS) and infection and injury of neurons [13]. After inoculation in the skin, WNV infects dendritic cell subsets and traffics to the draining lymph node (DLN) where it replicates locally and spreads to other secondary lymphoid and visceral organs [14]. In most human cases, WNV is cleared after induction of a robust innate and adaptive immune response. However, in the elderly, the virus crosses the blood-brain barrier at increased frequency and infects neurons of the brain and spinal cord; this is associated with a 5 to 10% case-fatality rate [14]. Based on animal studies, rapid clearance of WNV infection requires the concerted actions of multiple aspects of the immune system, including antiviral cytokine responses by myeloid cells [15], complement activation [16], the rapid induction of virus-specific neutralizing antibodies [17,18], helper, effector, and regulatory functions of CD4^+^ T cells [19–22], and priming of cytotoxic CD8^+^ T cells [23,24].

Analogous to humans, old mice are more vulnerable to WNV infection. One study reported a decreased number and function of WNV-specific CD8^+^ T cells [21], although the mechanistic basis of this defect was not defined. We hypothesized that age-dependent defects in B cell responses also contributed to disease susceptibility in old mice. Following WNV infection of young adult mice, an early T-independent and T-dependent IgM response develops, which limits viremia and prevents spread to the brain [17,18]. Subsequently, T-dependent class-switching and affinity maturation leads to the development of plasma cells that secrete higher affinity IgG antibodies, which neutralize virus in peripheral tissues [22]. Some of these antibody-secreting cells migrate to the CNS where they likely help to control and clear WNV infection [25].

In this study, we detected age-dependent defects in the early antigen-specific IgM and IgG response that correlated with increased viral titers in the serum, spleen and brain, and higher rates of WNV mortality. Using a series of adoptive transfer experiments coupled with two-photon intravital microscopy, we identified cell-intrinsic defects in naïve CD4^+^ T cells as well as reduced cytokine and chemokine levels in the DLN environment, both of which contributed to reduced cellular accumulation, delayed germinal center development, and immune responses. The cell-intrinsic defect was observed specifically in naïve CD4^+^ T cells and not in CD19^+^ B cells and was associated with an inability to migrate efficiently into and within the inflamed DLN. Thus, age-dependent immune defects that occurred within the first few days of WNV infection resulted in delayed immune cell recruitment, antigen recognition, and priming. These very early defects contributed to the failure to control WNV infection and prevent death.

## Results

### Old mice are more vulnerable to WNV infection and have reduced antiviral antibody responses

**Fig 1:**
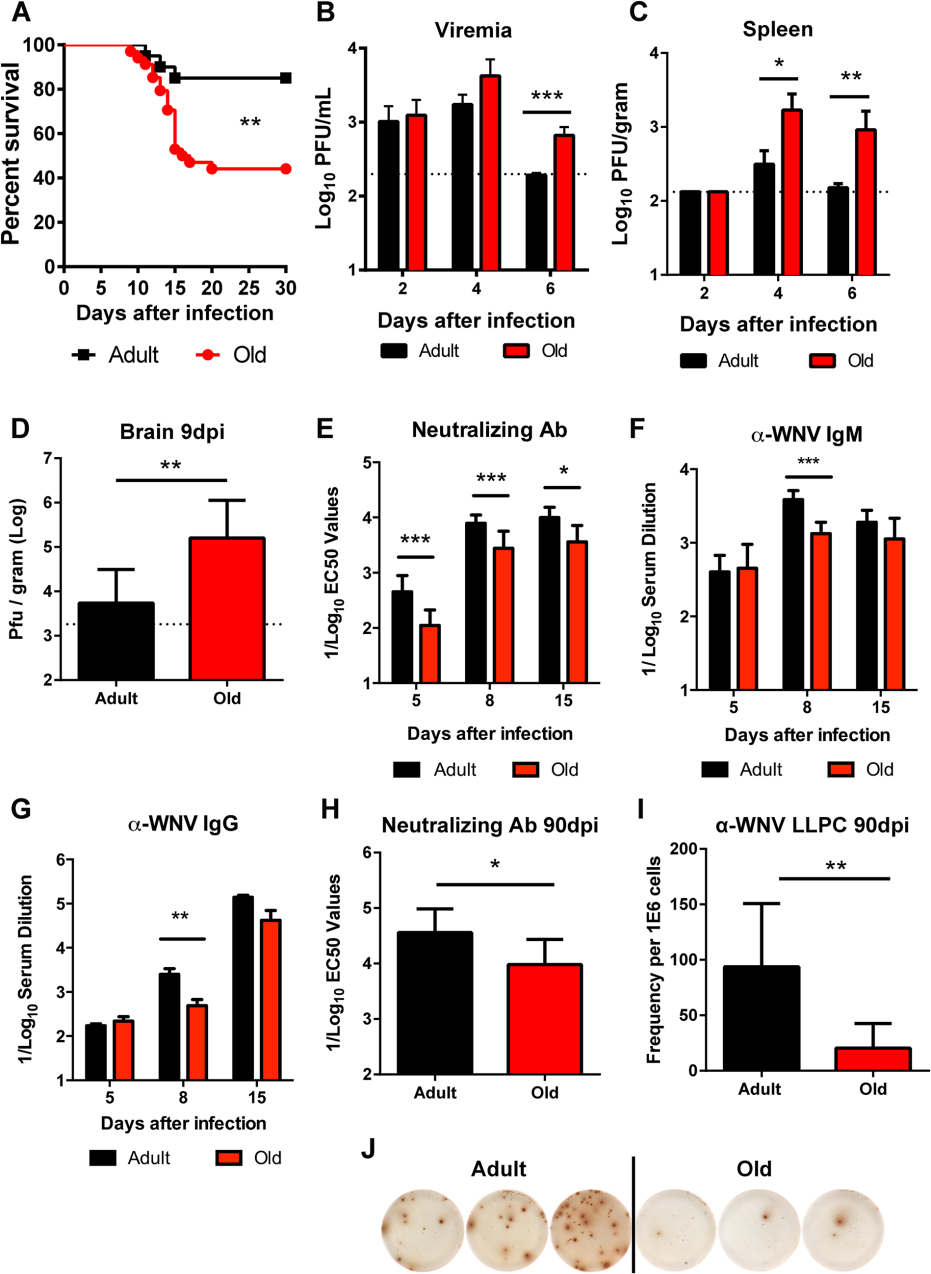
Survival rate, viral burden, and B cell responses after WNV infection. **A**. Adult or old mice were infected with 10^2^ PFU of WNV (New York strain) via subcutaneous injection in the footpad. Survival was monitored for 30 days: *n* = 20 adult (16 weeks) and *n* = 34 old (18 months) mice from at least three independent experiments. The difference was statistically different (*P* < 0.01; log rank test). **B-D.** Viral burden after WNV infection of adult and old mice was measured by qRT-PCR from serum (**B**) and plaque assay from spleen and brain (**C-D**). The data is shown as the mean titer (log_10_ PFU/ml) ± standard deviation (SD) and reflects 8 to 9 mice per time point from at least two independent experiments. The dashed lines represent the limit of sensitivity of the assay. Asterisks indicate statistical significance (*, *P* < 0.05; **, *P* < 0.01; ***, *P* < 0.001; Mann-Whitney test). **E-H**. Serum was obtained from adult and old mice at days 5, 8, 15, and 90 after WNV infection. **E and H**. Neutralizing antibody titers were determined by a focus-reduction assay. Results are shown as the mean of the reciprocal log_10_ titer ± SD that yields 50% inhibition of infection and reflects data from 5 to 11 mice per group from three independent experiments. **F-G**. Anti-WNV IgM (**F**) and IgG (**G**) levels were measured by ELISA. Data is plotted as the reciprocal log_10_ titer, defined as three times greater than background. **I-J.** Ninety days after infection, WNV-specific antibody secreting long-lived plasma cells in the bone marrow (LLPC) were quantified. Shown is the LLPC frequency per 10^6^ bone marrow cells ± SD (**I**), as well as representative ELISPOT images from three adult and three old mice (**J**). Data is pooled from 6 to 9 mice per group from three independent experiments.

We evaluated disease severity after WNV infection in adult and old mice. Compared to 4 month-old adult C57BL/6 mice, 18 month-old old syngeneic mice exhibited increased mortality (56% versus 15%, *P* < 0.01, **Fig 1A**) following subcutaneous infection with WNV (New York 2000 strain), corroborating results from a published report [21]. To understand the basis for the increased lethality, we measured viral burden in tissues. Older mice sustained increased levels of WNV in the serum at day 6 after infection (3.5-fold, *P* < 0.001 **Fig 1B**), in the spleen at days 4 (7-fold, *P* < 0.05) and 6 (17-fold, *P* < 0.01 **Fig 1C**) after infection, and in the brain at day 9 after infection (20-fold, *P* < 0.01 **Fig 1D**), which likely caused the higher mortality rate.

The early humoral immune response limits WNV infection and dissemination in adult mice [17,18]. We hypothesized that age-dependent defects in the WNV-specific humoral immunity might contribute to the more severe clinical phenotype in old mice. Accordingly, we assessed the WNV-specific antibody responses at days 5, 8, and 15 after infection in adult and old mice. Serum from old mice had less neutralizing activity than that from adult mice at all time points tested (2.7 to 4-fold, *P* < 0.05, **Fig 1E**). Also WNV-specific IgM and IgG antibody titers against the viral envelope (E) protein were lower at day 8 after infection in the aged mice (2.9 and 5.1-fold, *P* < 0.01, **Fig 1F and 1G**). By day 15, overall serum WNV E protein-specific IgM and IgG responses were equivalent in the surviving adult and old mice although a qualitative defect remained in the recognition of a dominant neutralizing epitope on domain III of the E protein [26,27] in old mice in both the IgM and IgG fractions (data not shown). Surviving old mice also had defects in durable humoral immunity, as their serum had reduced neutralization capacity at 90 days post infection (3.5-fold, *P* < 0.05, **Fig 1H**), which correlated with fewer WNV-specific long-lived plasma cells in the bone marrow (4.5-fold, *P* < 0.01 **Fig 1I and 1J**). Thus, old mice had delayed and decreased humoral immune responses during both the acute and memory phases after WNV infection.

### Decreased germinal center development in old mice

**Fig 2:**
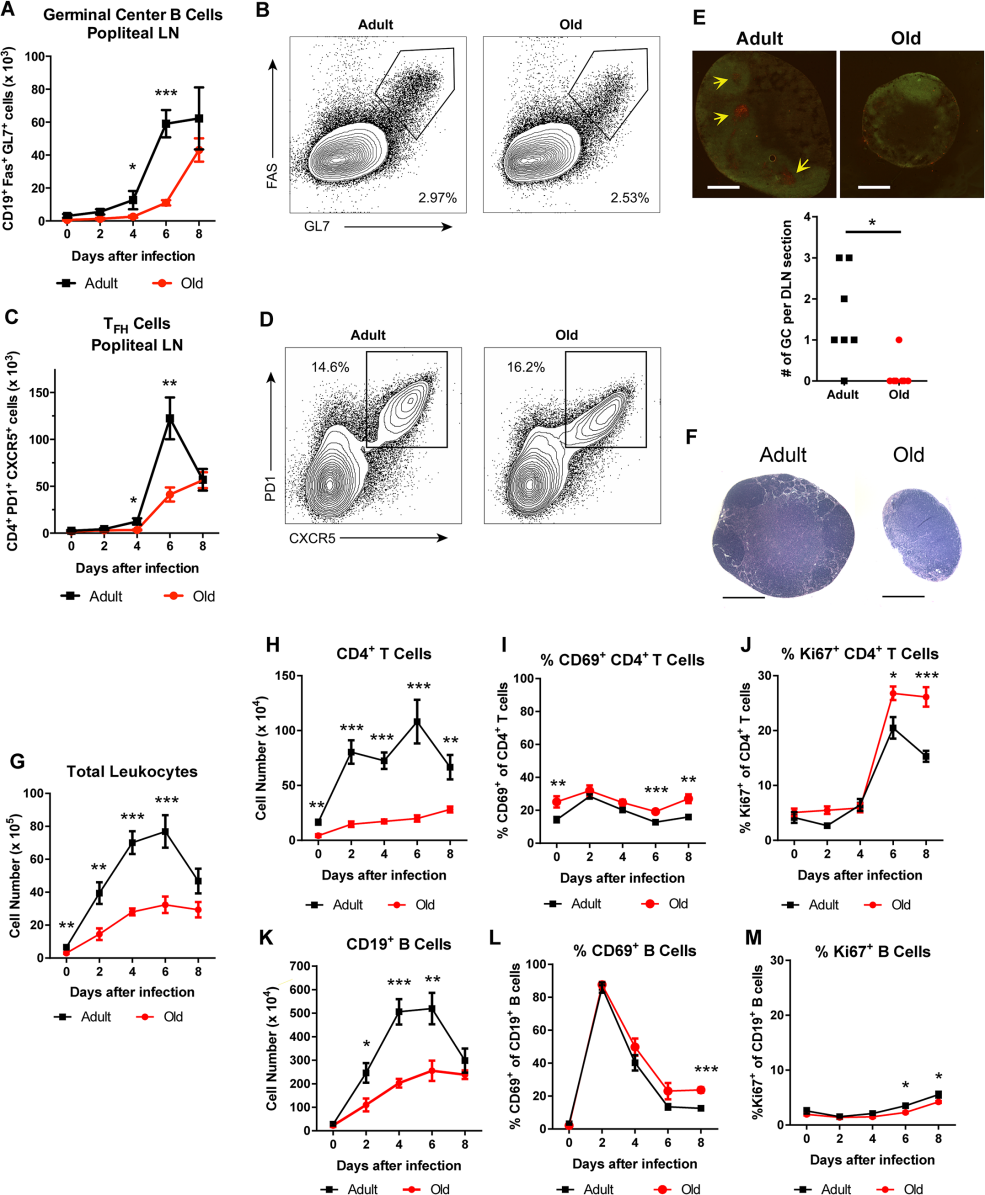
Delayed germinal center formation and decreased accumulation of CD4^+^ T cells and CD19^+^ B cells in DLN after WNV infection of old mice. Mice were infected with 10^2^ PFU of WNV (New York strain) in the footpad. **A-D**. Draining popliteal LN cells were analyzed by flow cytometry at the indicated days after infection. Numbers of GC B cells (CD19^+^ Fas^+^ GL7^+^ cells) (**A**) and T_FH_ cells (CD4^+^ PD1^+^ CXCR5^+^ cells) were quantified (**C**). **B and D.** Representative flow cytometry plots of GC B cells (**B**, on gated CD19^+^ cells) and T_FH_ cells (**D**, on gated CD4^+^ cells) from adult and old mice at 8 days post infection. **E**. Immunofluorescence staining of germinal centers in DLN from 6 days post WNV-infected adult and old mice. Slides were stained with GL7-PE, and IgD-FITC. Arrows point to sites of active GCs indicated by GL7 staining in the red channel. In the graph, the number of GCs per section is recorded with each point representing a section taken from the center of draining popliteal lymph nodes from adult or old mice from two independent experiments. **F.** Hematoxylin and eosin stained sections of the draining popliteal LN from adult and old mice 4 days post infection. **G-M**. At the indicated days post infection, the DLN was harvested, and total cells were counted (**G**). Antibody staining detected specific lymphocyte subsets including CD4^+^ T cells (**H**) and CD19^+^ B cells (**K**). CD4^+^ T cells (**I, J**) and CD19^+^ B cells (**L, M**) were co-stained with antibodies to CD69 (**I, L**) or Ki-67 (**J, M**) to establish their state of activation or proliferation. The results are pooled from at least three independent experiments with a total of 6 to 14 mice per group. Data is expressed as the mean ± the standard error of the mean (SEM). Asterisks indicate statistical significance (*, *P* < 0.05; **, *P* < 0.01; ***, *P* < 0.001; Mann-Whitney test).

To assess the basis for the defects in the early B cell response in WNV-infected old mice, we focused on the DLN. Following subcutaneous infection in the footpad, WNV traffics to the popliteal LN where an early B cell response is initiated [28]. Within the germinal centers of the DLN, T follicular helper (T_FH_) cells provide signals to germinal center B (GC B) cells to promote class switching and affinity maturation [29]. The accumulation of T_FH_ (CD4^+^ PD1^+^ CXCR5^+^) and GC B (CD19^+^ Fas^+^ GL7^+^) cells was decreased in old mice relative to adult mice at days 4 and 6, although nearly equivalent levels of T_FH_ and GC B cells were present at 8 days post infection (**Fig 2A and 2C**). These findings were corroborated by immunofluorescence microscopy analysis, which showed a reduced number of germinal centers at day 6 after infection (**Fig 2E**). The delayed germinal center development in the old mice matched the temporal pattern of antibody titers. At 6 days after infection, similar levels of CD28 were observed on CD4^+^ T cells and T_FH_ cells from old and adult mice (**S1A and S1B Fig**), suggesting that decreased expression of co-stimulatory molecules was not responsible for the delayed germinal center development in old mice. Consistent with this pattern, old mice had a proportionately higher number of CD4^+^ T cells and T_FH_ cells expressing the co-stimulatory molecule OX40 (CD134) (**S1C and S1D Fig**). T_FH_ cells from old mice did exhibit a small yet statistically significant reduction (11%, *P* < 0.01) in CXCR5 expression at 6 days post infection (**S1E Fig**). By 8 days after infection, however, a higher percentage of the CD4^+^ T cells from old mice were phenotypically T_FH_, and adult and old mice had equivalent numbers of GC B cells (**Fig 2A, 2B and 2D**). Thus, in old mice, the blunted humoral response to WNV correlated with a delay in the kinetics of germinal center development.

### Reduced lymphocyte accumulation in the DLN of old mice

During isolation of the popliteal lymph nodes for GC analysis, we noticed DLN from adult mice were substantially larger than those from old mice after WNV infection (**Fig 2E and 2F**). Because of this observation, we assessed how aging affected the total number of immune cells in the DLN at different time points after infection. In naive mice, we found increased cell numbers in the adult DLN relative to those from the old mice (6.6 x 10^5^ versus 3.0 x 10^5^ cells, *P* < 0.01 **Fig 2G**). After subcutaneous WNV inoculation, cell numbers increased rapidly in the adult mice, peaking at day 6. Cell accumulation was delayed in the DLN of old mice with fewer cells observed at days 2, 4, and 6 after infection (**Fig 2G**).

Cell subset analysis revealed that old mice had fewer CD4^+^ T and B cells in the DLN after WNV infection (**Fig 2H and 2K**), which could reflect reduced *in situ* proliferation and/or defects in cell trafficking. We monitored cell activation, as judged by surface acquisition of the marker CD69, and proliferation through expression of the nuclear protein Ki-67. As reported previously [28], CD19^+^ B cells in the DLN of adult mice were activated shortly after WNV infection with greater than 85% of cells expressing CD69 by day 2 (**Fig 2L**). The kinetics of CD69 up-regulation were equivalent on B cells in adult and old mice, although in old mice CD69 expression remained higher at day 8 (**Fig 2L** and data not shown). An analogous pattern of CD69 expression was seen in adult and old CD4^+^ T cells, although a smaller subset of cells was activated (e.g., 31% at 2 days after infection) (**Fig 2I**). We observed a similar small fraction of B cells and CD4^+^ T cells staining for Ki-67 in the adult and old mice between days 0 and 4 after infection (**Fig 2J and 2M**). An increase in Ki-67 expression was noted at day 6 for CD4^+^ T cells in both age groups, with higher levels in old mice at both days 6 and 8 after infection (**Fig 2J**). We also detected a small decrease in the frequency of Ki-67-positive proliferating B cells in old mice at days 6 and 8 after infection (3.5% to 2.3% at day 6 and 5.5% to 4.2% at day 8, *P* < 0.05, **Fig 2M**), which could reflect delays in the germinal center reaction observed in the old DLN (**Fig 2A and 2C**). These findings suggest that impaired activation or proliferation of lymphocytes did not account for the reduced cellular accumulation in the DLN of the old mice.

**Fig 3:**
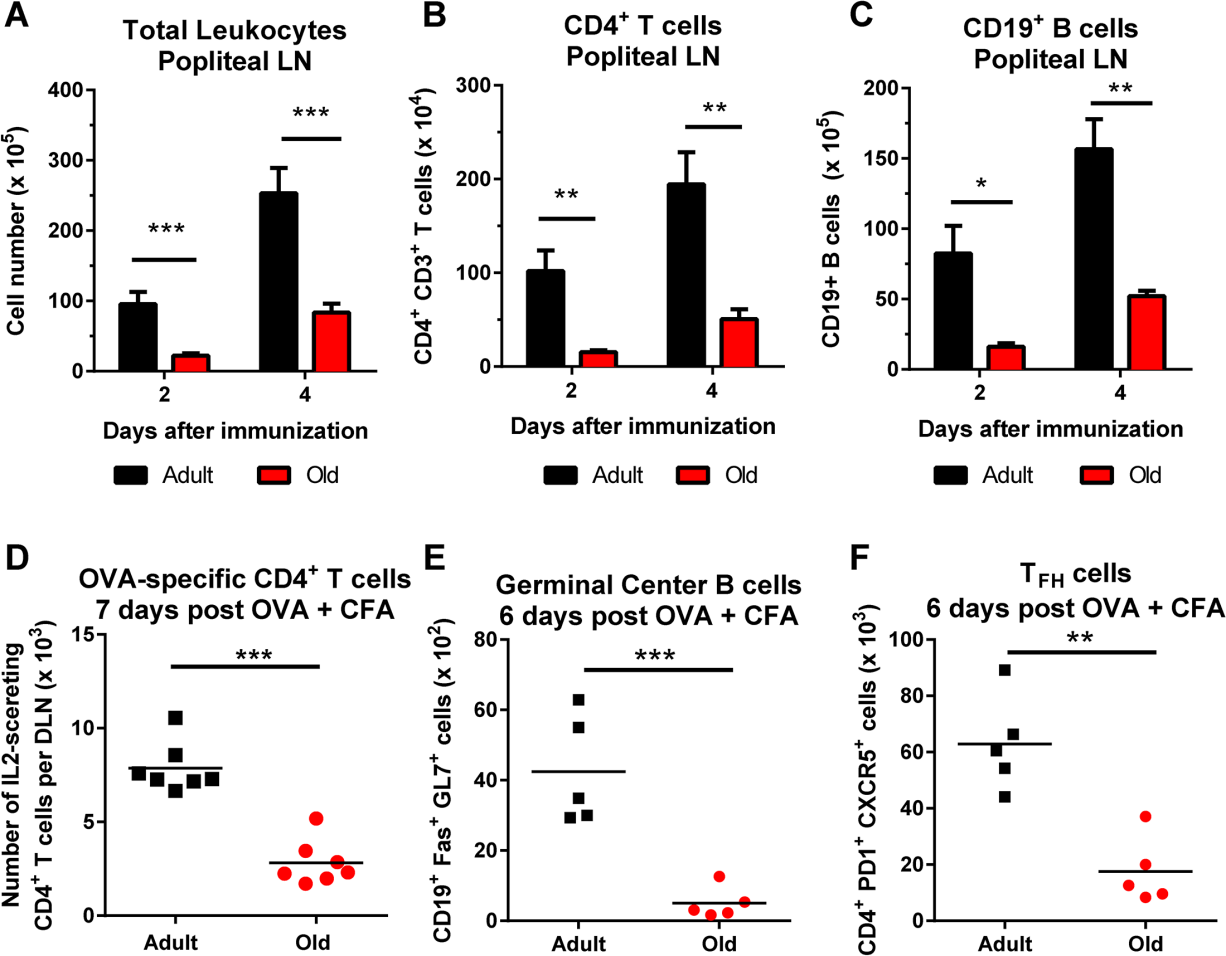
Decreased accumulation of CD4^+^ T cells and CD19^+^ B cells in DLN of old mice after immunization with ovalbumin. **A-C**. Adult and old mice were immunized with ovalbumin (OVA) complexed with complete Freund’s adjuvant in the footpad. At the indicated days post infection, the draining popliteal LN was harvested, and cells were counted (**A**). Antibody staining detected specific lymphocyte populations including CD4^+^ T cells (**B**) and CD19^+^ B cells (**C**). The results were pooled from two independent experiments with a total of 5 mice per group and data is expressed as the mean ± the standard error of the mean (SEM). **D**. Numbers of IL-2 secreting CD4^+^ T cells as judged by ELISPOT assay following OVA_323–337_ peptide stimulation in the DLN of adult and old mice at day 7 after immunization. **E-F.** Draining popliteal LN cells were analyzed by flow cytometry at 6 days after infection, and the numbers of GC B cells (CD19^+^ Fas^+^ GL7^+^) (**E**) and T_FH_ cells (CD4^+^ PD1^+^ CXCR5^+^) (**F**) were quantified. Each data point represents and individual mice and the results are pooled from two independent experiments. Asterisks indicate statistical significance (*, *P* < 0.05; **, *P* < 0.01; ***, *P* < 0.001; unpaired t test).

Diminished cellularity of the DLN of old mice was not unique to viral infection, as similar data was obtained with an inflammatory stimulus. Old mice immunized with ovalbumin emulsified in complete Freund’s adjuvant (CFA) had fewer CD4^+^ T cells and B cells in the DLN at early time points compared to adult mice (**Fig 3A–3C**). Old mice also had fewer IL-2-secreting antigen-specific CD4^+^ T cells at day 7 after immunization, and fewer germinal center B cells and T_FH_ cells at day 6 after immunization (**Fig 3D–3F**).

### Cell-intrinsic and cell-extrinsic defects in T cell recruitment into the DLN

**Fig 4:**
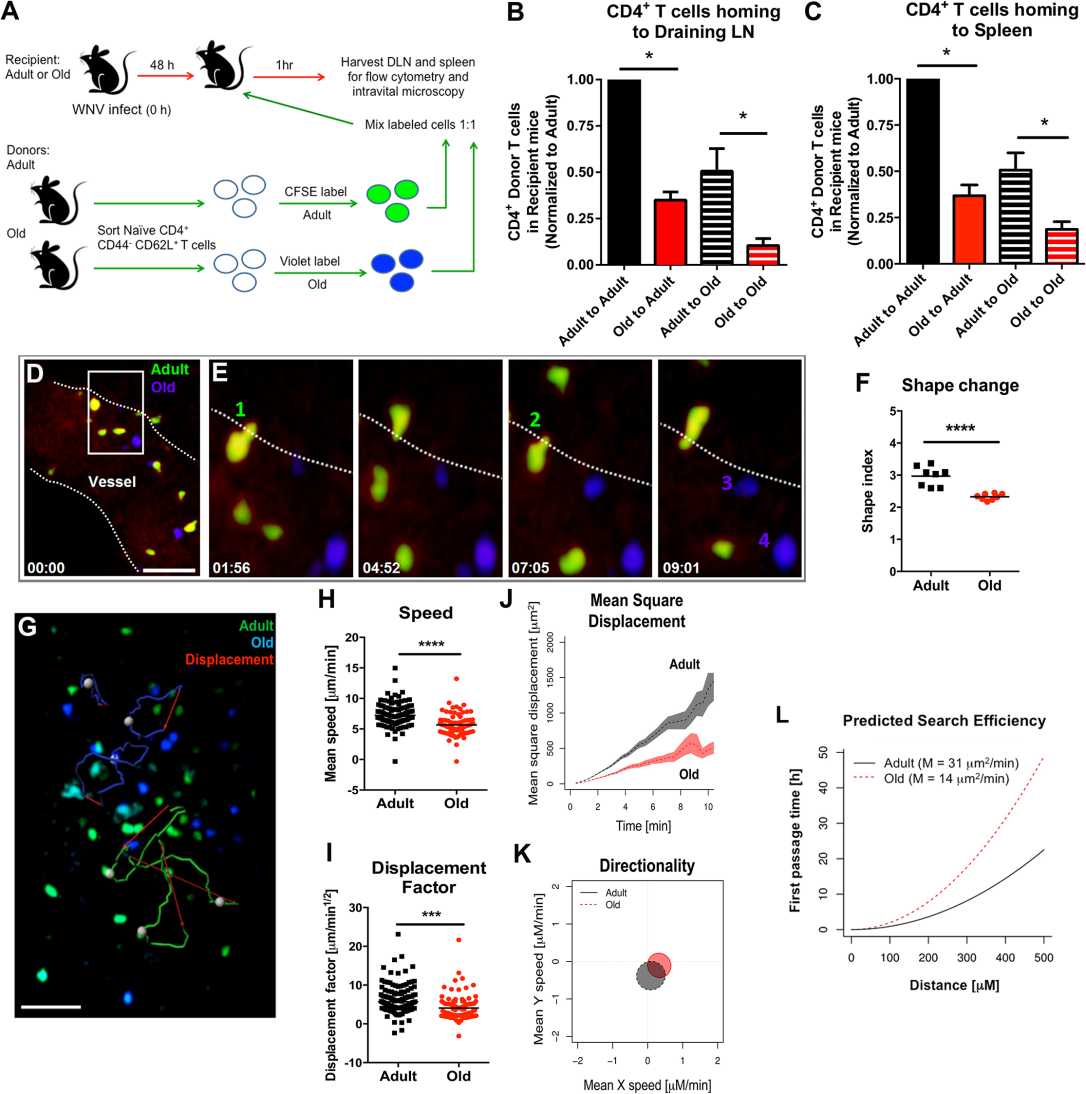
Defects in migration of naïve CD4^+^ T cells from old mice into inflamed LN. **A.** Scheme of adoptive transfer studies. 2 x 10^6^ FACS-sorted naïve (CD44^-^ CD62L^+^) CD4^+^ T cells from adult or old mice were differentially labeled with fluorescent dyes, mixed in a 1:1 ratio, and transferred to recipient adult or old mice that had been infected with WNV-KUN 48 hours earlier via a subcutaneous route. **B-C**. One hour later, draining popliteal LN (**B**) and spleen (**C**) were harvested. Cells were counted and the frequency of adult and old donor cells was determined by flow cytometry. In each tissue, the data was normalized to the levels of adult donor cells in the adult recipient organ and is a composite of four independent experiments. Asterisks indicate statistical significance (*, *P* < 0.05; Mann-Whitney test). **D-E**. Time-lapse two-photon intravital imaging of diapedesis of labeled donor naïve CD4^+^ T cells (adult = green; old = blue) in the HEV from the DLN of a recipient WNV-KUN-infected mouse. Images are individual frames from a continuous time-lapse movie (see **S1 and S2 Movies**). Relative time is displayed in min:sec. Panel **D** shows a picture of a Q-dot labeled vessel in a LN with multiple old (blue) and adult (green) CD4^+^ T cells adhering to the endothelium. The white dotted line is drawn to highlight the edge of the vessel lumen. The white box shows a zoomed in view of an extravasation "hot spot" that is then presented as a 4 time-lapse images in panel **E**. Two T cells (cells 1 and 2) from adult mice are shown undergoing diapedesis, whereas two representative T cells (cells 3 and 4) from old mice remain in the lumen over the same time frame. **F**. Cellular deformation of donor naïve CD4^+^ T cells in the HEV from the DLN of a recipient WNV-KUN-infected mouse as determined by analysis of two-photon microscopic images. **G.** Time-lapse two-photon imaging of movement of labeled donor naïve CD4^+^ T cells (adult = green; old = blue) in explanted DLN of a recipient WNV-KUN-infected mouse (see **S3 Movie**). The figure presents representative cell tracking for adult and old CD4^+^ T cells followed by evaluation of displacement. White opaque dots represent cells with tracked paths (green or blue). Red arrow indicates cell displacement. Scale bar (white): 40 μm. **H-L.** Analysis of movement parameters of adult and old donor naïve CD4^+^ T cells in explanted LN 6 to 8 hours post-transfer to recipient mice infected with WNV-KUN 48 hours earlier. Individual cells were tracked and (**H**) speed (μM/min), (**I**) cell displacement factor (μm/min^1/2^), (**J**) mean square cell displacement over time (μm^2^), (**K**) randomness of migration (Hotelling’s test of directionality) and (**L**) the predicted time of search efficiency for antigen. For panel **K,** the migration directionality was of a small magnitude, and no significant difference was observed between the cells from adult and old mice. For panel **L**, to judge the impact that the observed motility defects of old T cells would have on their ability to search antigen in DLN, we used a mathematical model [30] to predict the time needed by adult and old T cells to first reach increasingly distant locations (first passage time). The first passage time is proportional to the motility coefficient, which we estimated from the mean square displacement data. The predicted time to reach a location 500 μm away is ~20 hours for adult T cells, but ~50 hours for old T cells. The data in **F**, **H and I** are shown as a scatter plot and reflects three independent experiments. Asterisks indicate statistical significance (***, *P* < 0.001, ****, *P* < 0.0001; Mann-Whitney test).

We hypothesized that the reduced lymphocyte accumulation in the DLN of old mice soon after WNV infection or ovalbumin immunization could be due to cell-intrinsic defects in the lymphocytes and/or environmental defects in the DLN. To evaluate these possibilities, we sorted naïve CD4^+^ CD44^-^ CD62L^+^ T cells from lymphoid tissues of adult and old mice and labeled purified cells *ex vivo* with different fluorescent dyes (**Fig 4A**). As previously reported, old mice had fewer naïve CD4^+^ T cells compared to adult mice (**S2 Fig** and [5]). Equal numbers of differentially labeled adult and old naïve sorted CD4^+^ T cells were mixed at a 1:1 ratio and injected into recipient adult or old mice that had been infected two days earlier with the Kunjin strain of WNV (WNV-KUN), a less pathogenic variant that can be studied under A-BSL-2 conditions. Importantly, subcutaneous infection of old mice with WNV-KUN showed similar defects in cellular accumulation in the DLN compared to adult mice, with kinetics that mirrored that seen with the virulent WNV New York strain (**S3 Fig** and **Fig 2**). One hour after transfer of the labeled donor T cells into the WNV-KUN infected mice, the DLN of recipient mice were harvested. In both the adult and old recipients, greater numbers of adult donor T cells were detected within the DLN suggesting an intrinsic trafficking defect of the naïve CD4^+^ T cells from old mice (**Fig 4B**). We also observed reduced accumulation of adult and old donor cells in the old compared to adult recipients (**Fig 4B**), suggesting that the DLN environment separately contributed to the lower numbers of naïve CD4^+^ T cells recruited after infection. Similar defects were observed in the homing of cells to the spleen indicating that reduced trafficking could be a general property of naïve CD4^+^ T cells and the lymphoid environment of old mice (**Fig 4C**).

### Defects in migration of naïve CD4^+^ T cells

**Fig 5:**
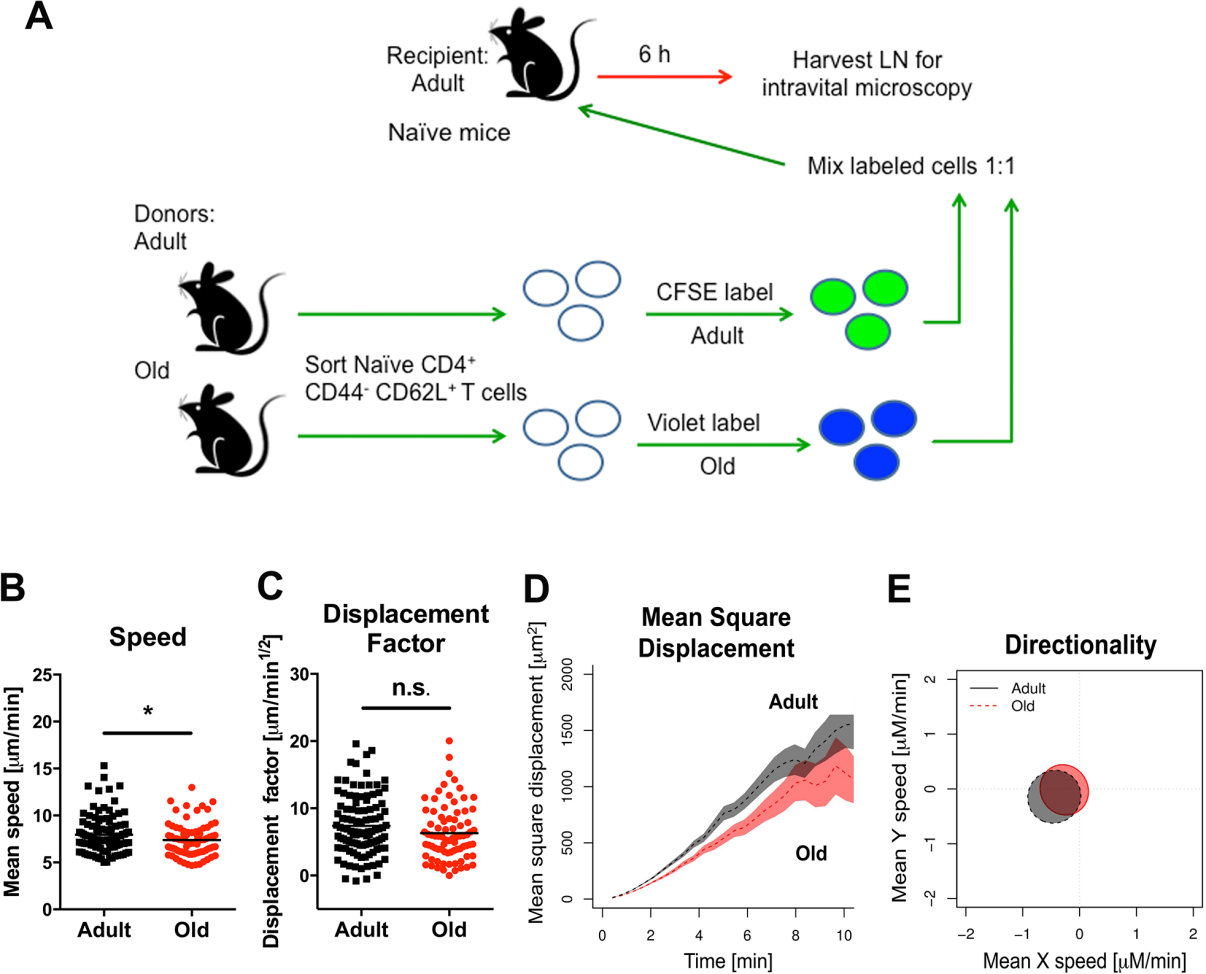
Migration of naïve CD4^+^ T cells from old mice into naive LN. **A.** Scheme of adoptive transfer studies of naïve CD4^+^ T cells. 2 x 10^6^ FACS-sorted naïve (CD44^-^ CD62L^+^) CD4^+^ T cells from adult or old mice were differentially labeled with fluorescent dyes, mixed in a 1:1 ratio, and transferred to recipient naïve adult mice. **B-E**. Six hours later, LN was harvested for microscopy studies. Analysis of movement parameters of adult and old donor naïve CD4^+^ T cells in explanted LN from recipient mice. Individual cells were tracked and (**B**) speed (μM/min), (**C**) displacement factor (μm/min^1/2^), (**D**) mean square cell displacement over time (μm^2^), and (**E**) directionality (Hotelling’s test) were measured. The data in **B-C** are shown as a scatter plot and reflects two independent experiments. Asterisks indicate statistical significance (*, *P* < 0.05; Mann-Whitney test).

We used intravital two-photon microscopy to evaluate the trafficking patterns of adult and old naïve CD4^+^ T cells *in vivo* within lymphoid tissues. Dye-labeled naïve CD4^+^ T cells from adult or old mice were mixed at a 1:1 ratio and adoptively transferred into adult recipient mice that had been infected with WNV-KUN two days prior. Donor cells from both adult and old mice efficiently rolled and arrested on the luminal side of the high endothelial venules (HEV) in the DLN of recipient infected mice (**Fig 4D and 4E, S1 Movie and S2 Movie**), although the appearance of donor T cells from old mice in the HEVs was delayed by ~15 minutes relative to donor cells from adult mice. Remarkably, naïve CD4^+^ T cells from old mice extravasated less efficiently compared to T cells from adult mice, with many cells unable to undergo the rapid cell shape deformation required for transendothelial migration across HEV (**Fig 4F**). This result suggested that although naïve CD4^+^ T cells from old mice could bind HEV, they were less efficient at entering the parenchyma of the DLN due to defective diapedesis. When the motility of extravasated T cells was analyzed in explanted lymph nodes from WNV-KUN infected adult recipient mice, adult donor naive CD4^+^ T cells moved with greater speed and displacement than the old donor naïve CD4^+^ T cells (**Fig 4G–4I, S3 Movie and S4 Fig**). However, neither adult nor old donor naive CD4^+^ T cells showed signs of chemotactically biased motion (**Fig 4K**). Computational analysis [30] predicted that the motility defects of naïve CD4^+^ T cells from old mice would delay encounter with antigen 500 μm away by ~30 hours (**Fig 4L**), which would contribute to the slowed kinetics of the humoral immune response in these animals. A trend towards reduced migratory capacity of old, compared to adult naïve CD4^+^ T cells also was observed in the LN of naive mice, although the magnitude of the difference was smaller (**Fig 5A–5E**).

Decreased expression of surface adhesion and chemoattractant receptors on naïve CD4^+^ T cells from old mice might explain the reduced transmigration and motility phenotype. However, when we assessed the expression of the surface adhesion molecules CD62L, PSGL1, LFA1, and VLA-4 as well as the chemokine receptors CCR7 and CXCR4 on naïve CD4^+^ T cells from adult and old mice, no significant differences in the geometric mean fluorescence intensity of expression of any of these molecules were observed (**S5 Fig**). We also did not find differences in the levels of filamentous actin in adult and old naïve CD4^+^ T cells following CCL19 (MIP-3β) and CCL21 (Exodus-2) stimulation (**S6A Fig**), suggesting that cytokine recognition, signaling through the Rac/Rho pathway, and actin polymerization/depolymerization were intact. Reduced T cell binding to ICAM-1 also could lead to diminished transmigration [31]. However, we observed no difference in the ability of naïve CD4^+^ T cells from adult or old mice to bind ICAM-1 coated substrates or migrate through ICAM-1 coated transwells towards CCL19 and CCL21 chemoattractants (**S6B and S6C Fig**).

### Age-dependent cell-intrinsic defects in naïve CD4^+^ T cells lead to reduced GC responses

**Fig 6:**
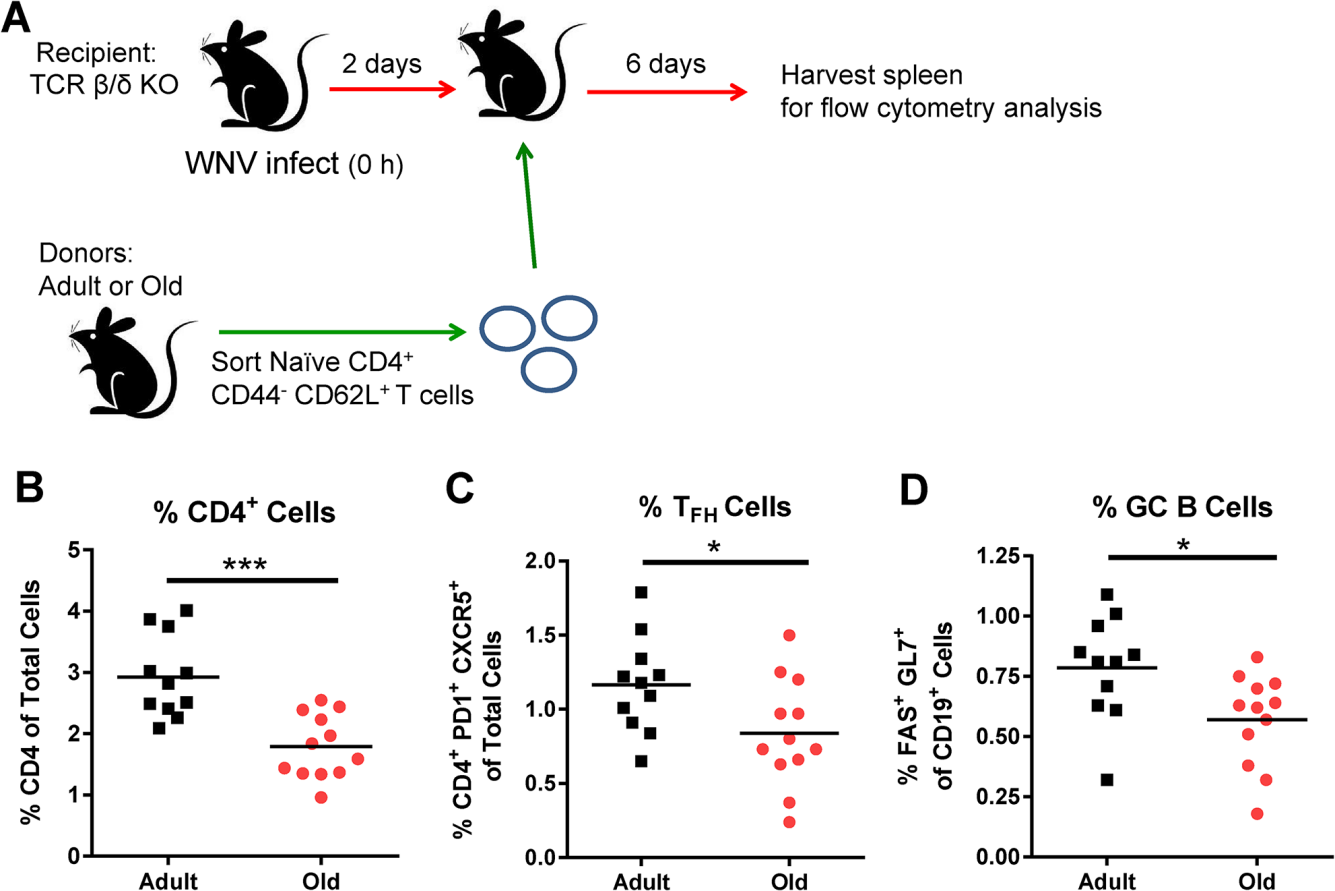
Transfer of naïve CD4^+^ T cells from old mice results in reduced GC responses. **A.** TCR β/δ^-/-^ recipient mice were infected with WNV (New York strain). Two days after infection, 3 x 10^6^ naïve sorted CD4^+^ T cells from adult or old mice were transferred adoptively into the recipient mice and 6 days post transfer (at 8 days post infection) spleens were analyzed for GC responses. Percentages of CD4^+^ T cells (**B**), T_FH_ cells (CD4^+^ PD1^+^ CXCR5^+^ cells) (**C**), and GC B cells (Fas^+^ GL7^+^ of CD19^+^ cells) (**D**) were quantified. The results are pooled from three independent experiments with a total of 11 to 12 mice per group, and each data symbol represents a single mouse. Asterisks indicate statistical significance (*, *P* < 0.05; ***, *P* < 0.001; unpaired t-test).

To link the reduced GC responses in old mice (**Fig 2**) with cell-intrinsic migratory defects of the naïve CD4^+^ T cells (**Fig 4**), we adoptively transferred sorted naïve CD4^+^ T cells from adult or old mice to recipient TCR β/δ ^-/-^mice (lacking T cells) that had been infected 2 days earlier with WNV (**Fig 6A**). We quantified CD4^+^ T cell and GC responses 6 days later at 8 days after infection. In all recipient TCR β/δ ^-/-^mice, very low GC responses were observed in the DLN, possibly because few antigen-specific CD4^+^ T cells reached this site (data not shown); however, GC responses were detected in the spleen. Animals receiving donor cells from old mice had a reduced percentage of CD4^+^ T cells in the spleen relative to those receiving adult donor cells (**Fig 6B**), and this corresponded with a lower percentage of T_FH_ and GC B cells (**Fig 6C and 6D**). These data suggest that intrinsic CD4^+^ T cell defects alone, independent of other age-dependent immunological defects, contribute to the reduced GC response following WNV infection in old mice.

### B cells from adult and old mice traffic to lymphoid tissues equivalently

**Fig 7:**
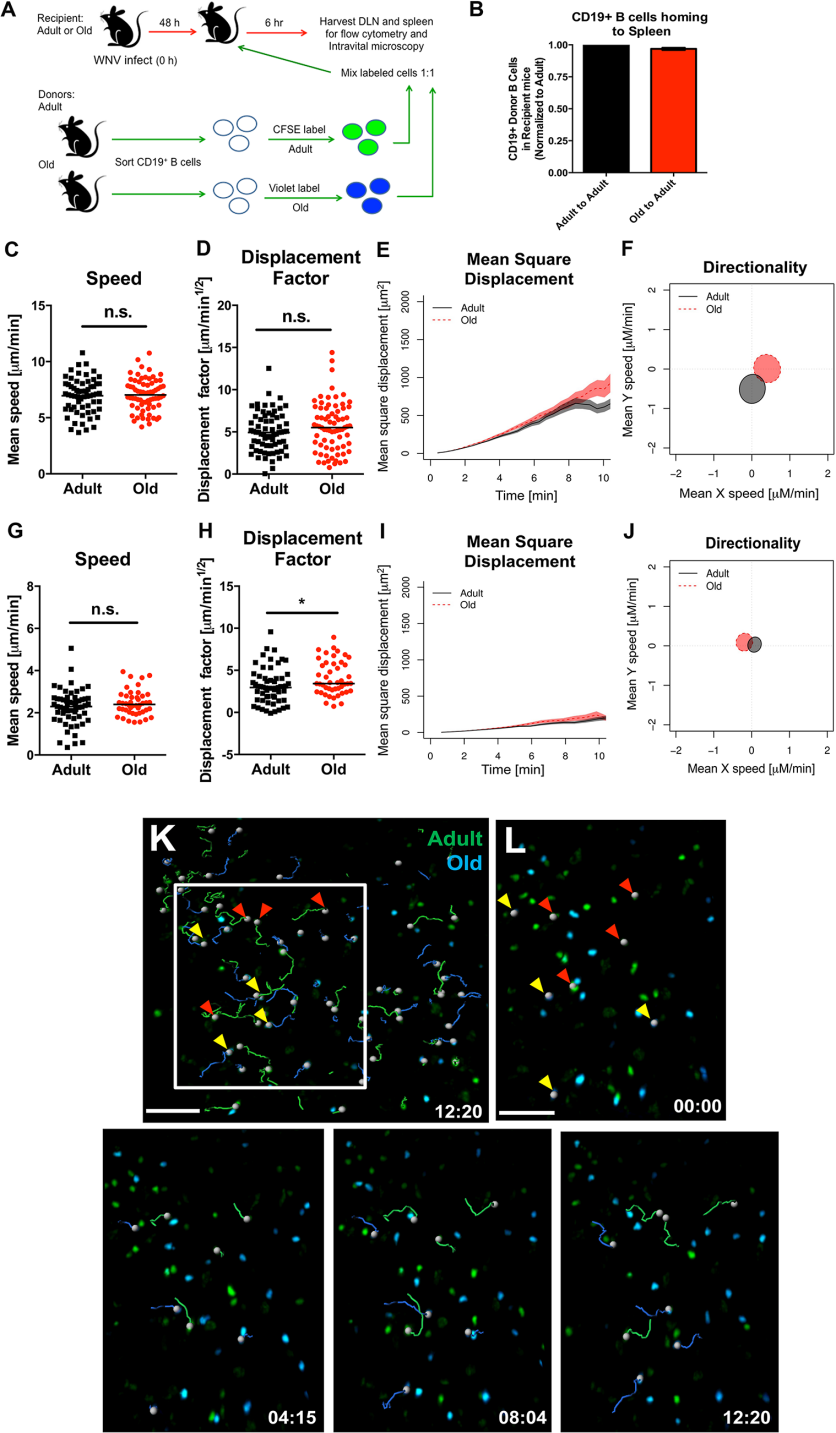
Normal migration of CD19^+^ B cells from old mice. **A.** Scheme of adoptive transfer studies of CD19^+^ B cells. 5 x 10^6^ isolated B cells from adult or old mice were differentially labeled with fluorescent dyes, mixed in a 1:1 ratio, and transferred to recipient adult mice infected with WNV-KUN 48 hours earlier via a subcutaneous route. **B**. Six hours later, spleens were harvested. Cells were counted and the frequency of adult and old donor cells was determined by flow cytometry. The data was normalized to the levels of adult donor cells in the recipient organ and is a composite of two independent experiments. **C-J**. Analysis of movement parameters of adult and old donor naïve CD19^+^ B cells in explanted LN (**C-F**) or spleen (**G-J**) 6 hours post-transfer to recipient mice that had been infected with WNV-KUN 48 hours earlier. Individual cells were tracked and (**C, G**) speed (μM/min), (**D, H**) displacement factor (μm/min^1/2^), (**E, I**) mean square displacement over time, and (**F, J**) directionality (Hotelling’s test) were determined. The data in **C, D, G, and H** are shown as a scatter plot and reflects two independent experiments. n.s. indicates the differences were not statistically significant; * indicates *P* < 0.05, Mann-Whitney test. **K-L.** Time-lapse image sequences of transferred adult and old CD19^+^ B cells in explanted LN in recipient mice 48 h after WNV-KUN infection. Differentially labeled (blue = old, green = adult) CD19^+^ B cells were adoptively transferred into WNV-KUN infected adult recipient mice (48 h after infection) and DLN were harvested 6 to 8 h later followed by ex vivo imaging. Panel **K** presents all tracked adult and old cells during the first 12 minutes within the cropped area with the white frame. Panel **L** and others presents time-lapse image sequences of adult and old cell tracking during first 12 minutes. Yellow and red arrows indicate which old and adult cells were analyzed and their starting points, respectively. Scale bar is indicated in white. Images are individual frames from a continuous time-lapse movie (**S4 Movie**).

We next tested whether B cells also had a trafficking defect by adoptively transferring a 1:1 ratio of differentially dye-labeled adult and old B cells into adult recipient WNV-KUN infected mice and quantifying cell recruitment. No major differences were observed in the numbers of adult and old donor B cells in lymphoid tissues at 6 hours after transfer (**Fig 7A** and **7B**), suggesting that impaired cell accumulation was not a general phenomenon that affected all lymphocyte subsets from old mice. Similar results were obtained in the spleen at 1 hour after transfer (data not shown). CD19^+^ B cells also did not show defects in migration in explanted lymph nodes (**Fig 7C–7F and 7K and 7L**) or spleen (**Fig 7G–7J and S4 Movie)**.

**Fig 8:**
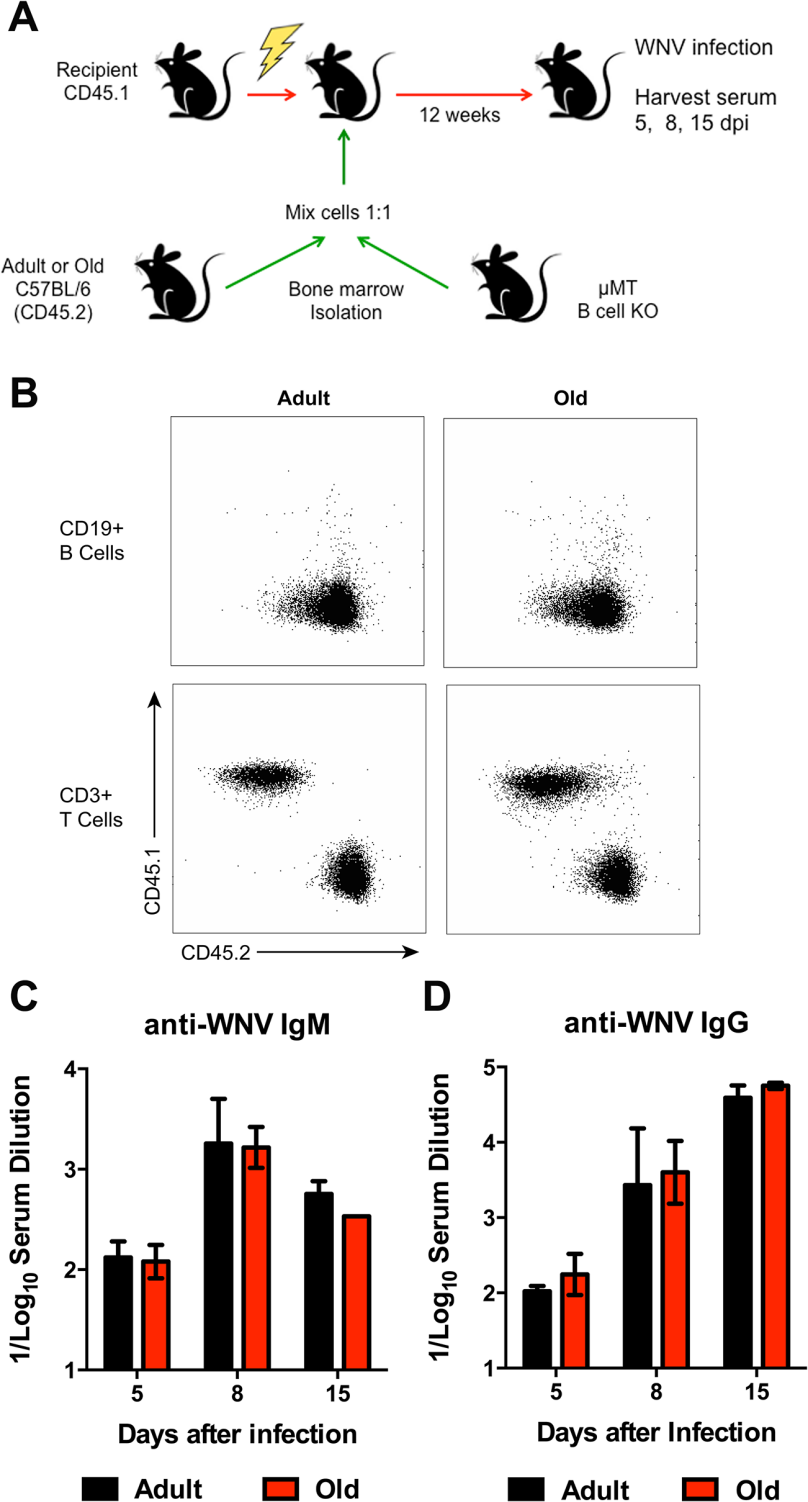
Humoral immune response of donor bone marrow into irradiated recipient mice. **A.** Scheme of reconstitution studies with bone marrow from adult or old mice. 10^7^ bone marrow cells from adult or old mice were mixed with 10^7^ bone marrow cells from μMT (B cell deficient) mice and transferred into irradiated recipient CD45.1 mice. After 12 weeks, mice were infected with WNV (New York strain) and serum was harvested 5, 8, and 15 dpi. **B.** After 12 weeks post transfer, blood was sampled and tested for reconstitution efficiency. The vast majority (>95%) of circulating CD19^+^ B cells was derived from the CD45.2 donor mice. **C-D**. IgM (**C**) and IgG (**D**) levels were measured by ELISA for reactivity with WNV E protein. Data is plotted as the reciprocal log_10_ titer and represents data from 5 mice per group.

To further assess possible cell-intrinsic defects in B cells from old mice we adoptively transferred an equal number of bone marrow cells from adult or old CD45.2 mice into separately irradiated CD45.1 adult recipient mice. We also transferred bone marrow cells from adult B cell-deficient (μMT) to provide equivalent T cell help (**Fig 8A**). Twelve weeks later, the transfers were confirmed by analyzing circulating CD19^+^ B cells in blood (**Fig 8B**). Recipient mice were infected with WNV and serum was harvested for evaluation of WNV-specific antibody responses at days 5, 8, and 15 after infection. Notably, recipient mice reconstituted with B cells from adult or old mice had similar WNV-specific IgM and IgG responses (**Fig 8C** and **8D**).

### Reduced leukocyte migration and chemokines in the DLN

**Fig 9:**
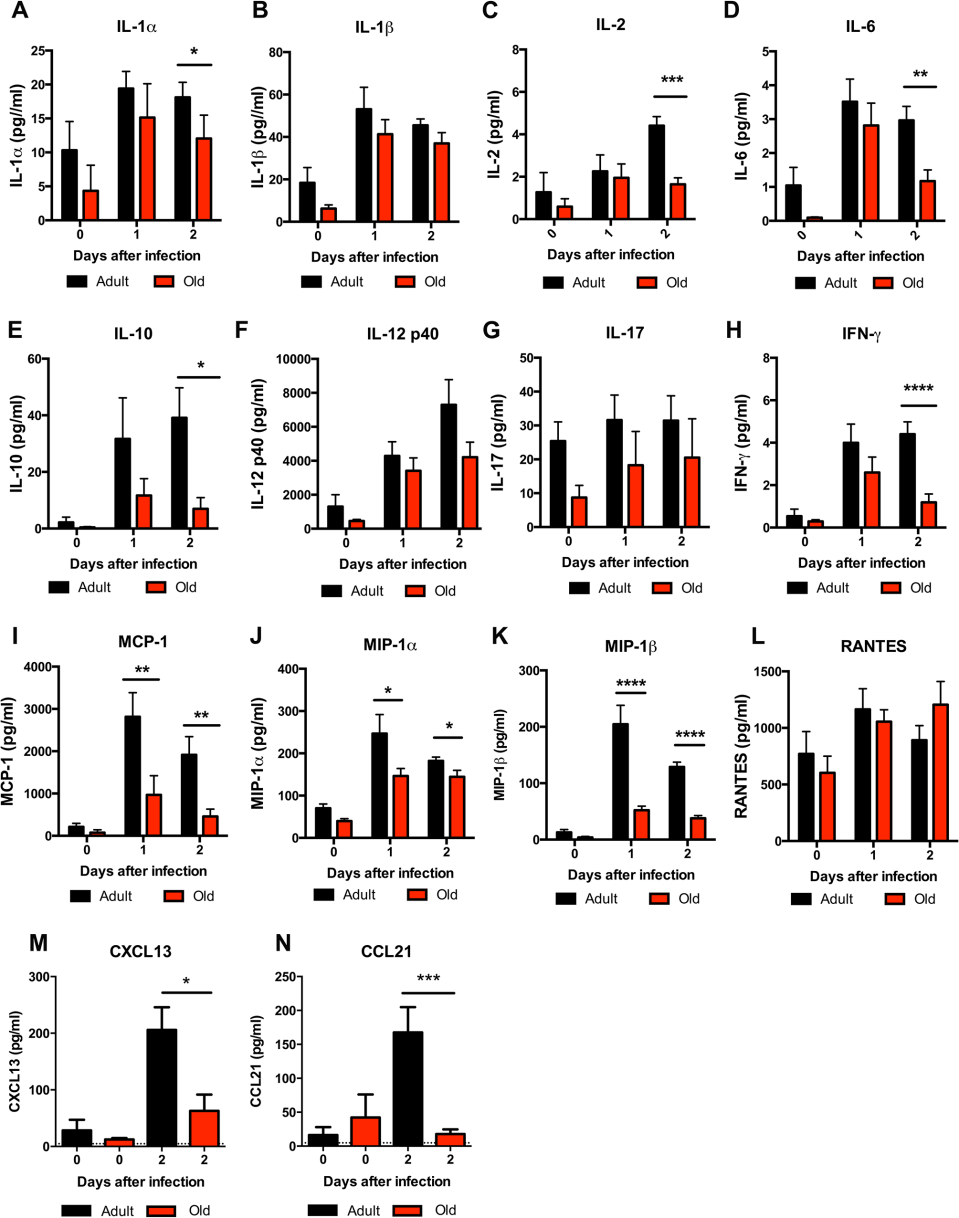
Decreased levels of cytokines and chemokines in the DLN of old mice. Adult and old mice were infected bilaterally with 10^2^ PFU of WNV (New York strain) in the footpad and DLN were harvested at days 0, 1, and 2 after infection. **A-L**. Levels of cytokines (IL-1α (**A**), IL-1β (**B**), IL-2 (**C**), IL-6 **(D**), IL-10 (**E**), IL12 p40 (**F**), IL-17, (**G**), and IFN-γ (**H**)) and chemokines (MCP-1 (**I**), MIP-1α (**J**), MIP-1β (**K**), and RANTES (**L**)) in DLN were measured by Bio-Plex assay. **M-N.** Levels of chemokines (CXCL13 (**M**) and CCL21 (**N**)) in DLN were measured by ELISA. Data are presented as mean + SEM and reflects a total of 6 to 8 mice for each group from two independent experiments. Asterisks indicate statistical significance (*, *P* < 0.05; **, *P* < 0.01; ***, *P* < 0.001; ****, *P* < 0.0001; Mann-Whitney test).

**Fig 10:**
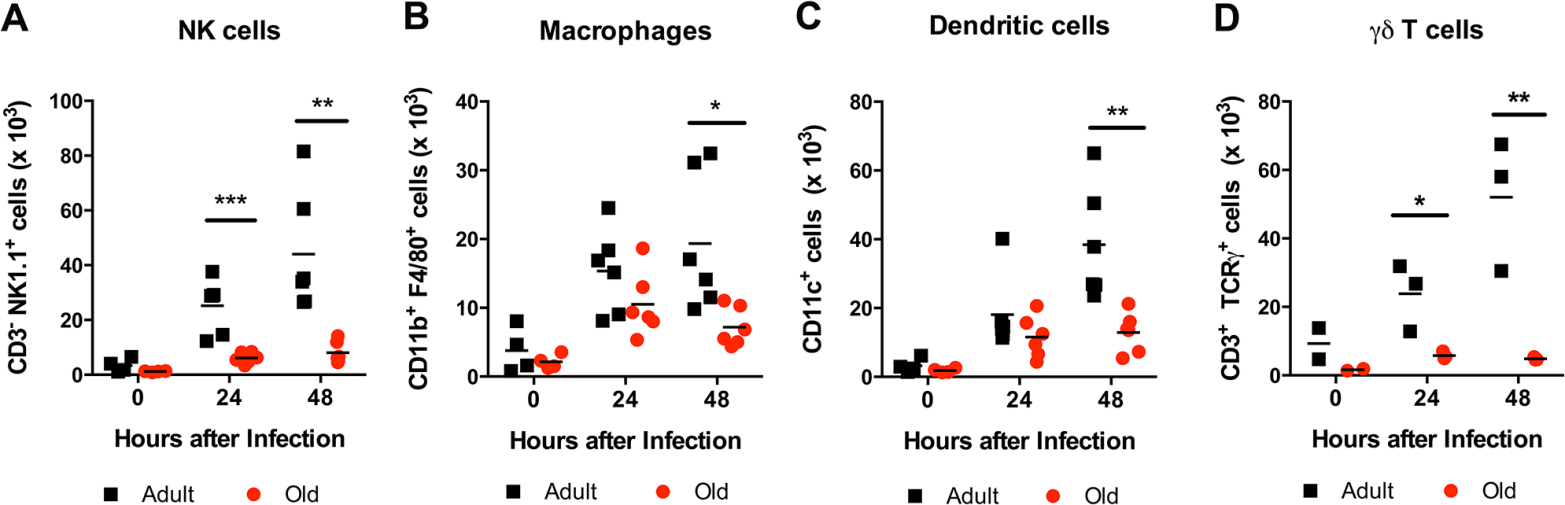
Leukocyte infiltration in the DLN of WNV-infected adult and old mice. Adult and old mice were infected with 10^2^ PFU of WNV (New York strain) in the footpad. One or two days later, the draining popliteal LN was harvested, cells were stained with antibodies to detect specific cell populations including (**A**) NK1.1^+^ NK cells, (**B**) CD11b^+^ F4/80^+^ macrophages, (**C**) CD11c^+^ dendritic cells, and (**D**) γδ T cells. The results are depicted as a scatter plot and are pooled from two independent experiments with a total of 6 mice per group (with the exception of γδ T cells, which reflects 3 mice per group). Asterisks indicate statistical significance (*, *P* < 0.05; **, *P* < 0.01; ***, *P* < 0.001; Mann-Whitney test).

The reduced accumulation of adult or old donor cells in old recipient lymphoid tissue (**Fig 4B and 4C**) suggested a separate environmental defect independently contributed to the reduced migration of naïve CD4^+^ T cells. To address the basis for these findings, we measured the levels of pro-inflammatory cytokines and chemokines in the inflamed DLN (**Fig 9**). Within one to two days of WNV infection, lower levels of several chemokines were apparent in the homogenates of DLN from old mice. This included chemoattractants for monocytes (MCP-1 (CCL2)), granulocytes (MIP-1α (CCL3)), NK cells (MIP-1β (CCL4)), B cells (BLC (CXCL13)), and was associated with the subsequently decreased production of proinflammatory cytokines including IL-1α, IL-2, IL-6, and IFN-γ, and the anti-inflammatory cytokine IL-10. Relevant to our naïve CD4^+^ T cells findings, the DLNs from old mice had nearly a 10-fold decrease in the naïve T cell chemoattractant CCL21 at day 2 after infection (**Fig 9N**). Consistent with the blunted production of chemokines, we also observed diminished accumulation of NK cells, γδT cells, macrophages, and dendritic cells in the DLN of old mice after WNV infection (**Fig 10**). Thus, in addition to cell-intrinsic defects of naïve CD4^+^ T cells in old animals, the DLNs of old mice separately have attenuated inflammatory cytokine and chemokine responses, which reduced the recruitment of other cell types that orchestrate adaptive immunity against WNV infection.

## Discussion

The decline of the immune system with age correlates with increased susceptibility to many infectious diseases and a decreased response to vaccines. In this study, using a mouse model of aging in the context of WNV infection, we identified cell-intrinsic defects in naïve CD4^+^ T cells as well as environmental defects that resulted in delayed and reduced lymphocyte accumulation in the inflamed DLN of old mice at very early time points. These defects delayed the development of the early germinal center reaction, which resulted in blunted humoral immune responses that likely contributed to increased viral titers in the blood and peripheral organs and enhanced vulnerability to lethal infection. Even small differences in WNV-specific antibody levels at early times after infection affect dissemination to the brain and clinical outcome [17,18]. As mice lacking B cells uniformly succumb to WNV infection [17], the delayed adaptive immune response in old mice still provided a significant level (~50% survival) of protection. Old mice surviving the acute stage of infection eventually developed similar overall levels of WNV-specific antibody titers and germinal center B and T_FH_ cells compared to adult mice, although qualitative defects in the humoral immunity remained. In addition, old mice had deficits in their long-lived plasma cell memory response relative to the adult mice. This suggests that despite GC B cells and T_FH_ cells reaching equivalent levels in the surviving adult and old mice after WNV infection, functional defects persist in the germinal center reactions of old mice, which contribute to blunted memory responses. While further study is warranted, part of the age-dependent differences in memory phenotypes could be associated with differential expression of pro-survival (e.g., Bcl-2) or pro-apoptotic (e.g., TRAIL) proteins in B cells [32,33] or less likely the lower levels of CXCR5 observed on T_FH_ cells.

Although the association between aging and susceptibility to viral infection has been described [34,35], our results support a model in which immune system deficits occurring shortly after infection determine clinical outcome. Within 48 hours of WNV infection, we detected a diminished immune response in the DLN of old mice that included reduced accumulation of NK cells, dendritic cells, macrophages, and B and T cells. Associated with this phenotype were decreased levels of several cytokines and chemokines, including the naïve T cell chemoattractant CCL21 and the B cell chemoattractant CXCL13. These results are consistent with reports showing disrupted cytokine production after pattern recognition receptor stimulation in innate immune cells from old mice and humans [3]. Defects in the cellular production of cytokines and chemokines in the LN of old mice likely contribute to the reduced accumulation of innate and adaptive immune cells after inflammatory or infectious stimuli. Consistent with this idea, reduced levels of CCL19 and CCL21 were detected in the spleen of old mice immunized with OVA absorbed on alum [36]. These chemokine and recruitment defects also may lead to defective priming of CD8^+^ T cells, as the DLN has an important role in priming these cells during a systemic viral infection [37]. Thus, our data provides an explanation why qualitative and quantitative defects in antigen-specific CD8^+^ T cells responses also exist after WNV infection of old mice [21].

Prior studies have reported reduced frequencies of naïve CD4^+^ T cells in old animals, similar to our observations, due to long-term antigen exposure, conversion to memory phenotypes, and repertoire contraction [5]. We also observed a separate, independent cell-intrinsic defect in the naïve CD4^+^ T cells from old mice. By adoptively transferring adult or old naïve CD4^+^ T cells into recipient mice lacking T cells, we found that intrinsic defects in the naïve CD4^+^ T cells of the old mice contributed to reduced GC responses independent of other factors, analogous to previous immunization models with transgenic donor cells [10]. Although others have identified naïve CD4^+^ T cell-intrinsic defects following antigen encounter [38], we identified an earlier, age-dependent deficit in the migratory capacity of the naïve CD4^+^ T cells. Soon after adoptive transfer of naïve CD4^+^ T cells from adult and old donors into adult recipient mice, cells from the old mice accumulated in DLNs to lower levels as compared to cells from adult mice. Intravital microscopy revealed that the trafficking of naïve CD4^+^ T cells from old mice was impaired at the diapedesis step specifically, as cell rolling and adhesion in HEVs was delayed only slightly relative to cells from adult mice. The reduced trafficking of naïve CD4^+^ T cells to DLN in old mice suggests that immune surveillance declines during aging. Once naïve CD4^+^ T cells from old mice entered the parenchyma of the inflamed LN, we also observed reduced motility. Although the T cell trafficking defects may seem modest, mathematical modeling [30] predicted that the reduced motility in the DLN could lead to many hours of delay in finding cognate antigen. Combined with the additional homing defects, this would hinder the induction of antigen-specific adaptive immune responses substantially. Due to the rapid nature of viral replication and dissemination, such a lag could and likely does alter disease outcome.

What is the cell-intrinsic defect of naïve CD4^+^ T cells from old animals that results in a reduced migratory capacity? A diminished capacity of naïve CD4^+^ T cells from old animals to activate and proliferate following TCR stimulation has been reported [38]; this has been ascribed to reduced cytoskeletal rearrangement [39,40], aberrant surface glycosylation [41], and altered phosphorylation of key signaling molecules [42]. These defects in naïve CD4^+^ T cells from old animals can be overcome to some extent by cytokine (e.g., IL-2) stimulation [43] or treatment with O-sialoglycoprotein endopeptidases [44]. We found no differences in the expression of cell-surface molecules important for chemokine binding or migration in adult or old mice. We also did not detect differences in the ability of naïve CD4^+^ T cells from adult and old mice to bind ICAM-1 or undergo cytoskeletal rearrangement after chemokine activation. Instead our studies suggest a key cell-intrinsic migratory defect in naïve CD4^+^ T cells from old mice that occurs *in vivo* prior to antigen stimulation at very early times relative to infection. Impaired function of non-muscle myosin IIA in CD4^+^ T cells from elderly humans and old mice has been linked to defective migration *ex vivo* [40] and an inability to deform nuclei [45] respectively, which are processes critical to successful diapedesis; however, we found no difference in the ability of naïve CD4^+^ T cells from adult and old mice to migrate across an ICAM-1-substrate *ex vivo*. Alternatively, mTOR inhibition rescues some of the age-related functional defects in naïve CD4^+^ T cells from old mice, suggesting that a reduced metabolic capacity could affect energy stores [44,46]. As migratory activities require extensive energy-dependent cytoskeletal rearrangements, generation of leading edges and uropodia, lower energy reserve levels could compromise this process.

In comparison to naïve CD4^+^ T cells, and even though humoral responses were blunted, we did not observe significant defects in the homing, motility, or function of CD19^+^ B cells from old mice. Intravital microscopy in explanted lymphoid tissues showed similar movement and displacement by B cells from old and adult mice. Moreover, in experiments in which bone marrow from old and adult mice were transferred into irradiated adult recipient mice and then infected with WNV, differences in the antiviral antibody response were not apparent; this confirms previous reports suggesting that newly derived B cells are functionally more intact than long-lived B cells in old animals [47]. These findings suggest that the defects in the naïve CD4^+^ T cells from old mice are not associated with a general functional decline of all lymphocyte subsets.

In summary, using a mouse model of WNV infection, we identified a specific age-dependent decline in the trafficking of naïve CD4^+^ T cells. Efficient T cell trafficking is required for optimal antigen recognition in the DLN and for timely initiation of T-helper dependent antibody induction. Because the rapid production of neutralizing and inhibitory antibodies is essential for limiting WNV replication and dissemination [17,18], even a modest delay in the kinetics of the humoral response in elderly persons could increase susceptibility to virus infection. As functional deficits in the cells that coordinate early adaptive immune response and the LN microenvironment exist, strategies to correct these defects in at-risk individuals could improve responses to vaccines and limit replication of microbes that promote rapid disease pathogenesis.

## Materials and Methods

### Ethics statement

This study was carried out in strict accordance with the recommendations in the Guide for the Care and Use of Laboratory Animals of the National Institutes of Health. The protocols were approved by the Institutional Animal Care and Use Committee at the Washington University School of Medicine (Assurance Number: A3381-01). Dissections and footpad injections were performed under anesthesia that was induced and maintained with ketamine hydrochloride and xylazine, and all efforts were made to minimize suffering.

### Reagents and antibodies

Cell Trackers: Cell Tracker Orange CMTMR (5-(and-6)-(((4-Chloromethyl) Benzoyl) Amino) Tetramethylrhodamine), CellTrace CFSE (carboxyfluorescein diacetate, succinimidyl ester) and CellTrace Violet were purchased from Life Technologies. The following fluorochrome or biotin-conjugated antibodies were purchased from BD Biosciences, Biolegend, and eBioscience (clone name in parenthesis): CD3ε (145-2C11), CD4 (RM4-5), CD8α (53–6.7) CD11b (M1/70), CD11c (HL3), CD19 (1D3), CD28 (E18), CD44 (IM7), CD45R (RA3-6B2), CD62L (MEL-14), CD69 (H1.2F3), OX40/CD134 (OX-86), Ki67 (SolA15), PD1 (29F.1A12), FAS (Jo2), GL7, F4/80 (BM8), TCRγ (eBioGL3), NK1.1 (PK136), VLA4 (R1-2), LFA-1 (2D7), PSGL-1 (2PH1), CCR7 (4B12), CXCR4 (L276F12), and CXCR5 (2G8).

### Viruses and cells

The WNV strain (3000.0259) was isolated in New York in 2000 and passaged once in C6/36 *Aedes albopictus* cells. The WNV-KUN strain (CH16532) was a gift of R. Tesh (World Reference Center of Emerging Viruses and Arboviruses, Galveston, TX) and amplified as described previously [48]. Viral titer was determined by plaque assay using Vero cells, as described previously [49].

### Mouse experiments

C57BL/6 mice were purchased from The Jackson Laboratory (4 month-old) (Bar Harbor, ME) or acquired from the National Institute of Aging dedicated breeding colony at Charles River Laboratories (18 month-old) (Wilmington, MA). TCR β/δ^-/-^ mice were purchased from The Jackson Laboratory and bred in the animal facilities at the Washington University School of Medicine. All mice were housed in a pathogen-free mouse facility at the Washington University. Mice were inoculated subcutaneously via footpad injection with 10^2^ plaque-forming units (PFU) of WNV-NY or 10^4^ PFU of WNV-KUN diluted in 50 μl of Hanks balanced salt solution (HBSS) supplemented with 0.1% heat-inactivated fetal bovine serum (FBS). For intravital imaging of the draining cervical lymph node, mice were infected subcutaneously in the nape of the neck.

### Measurement of viral burden

At specified time points after WNV infection, serum was obtained by intracardiac heart puncture, followed by intracardiac perfusion (20 ml of PBS), and organ recovery. Organs were weighed, homogenized using a bead-beater apparatus, and titrated by plaque assay on BHK21-15 cells as described previously [49].

### Bone marrow chimera and transfers

Bone marrow cells were collected from adult or old C57BL/6 (CD45.2) mice and adult B-cell deficient μMT mice. Adult or old bone marrow cells were mixed with an equal number of μMT bone marrow cells and 1 x 10^7^ cells were transferred adoptively by retroorbital injection into 8 to 12-wk-old 800 cGy–irradiated B6.SJL (CD45.1) mice (National Cancer Institute). Twelve weeks later, reconstitution was validated by flow cytometry and mice were infected with WNV.

### Ovalbumin immunizations

Mice were injected subcutaneously in the footpad with an emulsion containing CFA and 10 nmoles of OVA_323–337_ peptide, and DLN were analyzed at indicated time points. The ELISPOT assay has been described previously [50,51]. To evaluate MHC class II specific T cell responses in adult and old mice, 5 x 10^5^ cells from the DLN were harvested at day 7 post-immunization and plated on 96-well plates with Immobilon-P membrane coated with anti-mouse IL-2 antibody. After overnight incubation, plates were washed, incubated with a secondary antibody and developed as previously described [50,51]. Spots were analyzed on dedicated equipment and software (Cellular Technology Ltd along with Immunospot software Version 5.0.9) to calculate the total number of antigen-specific CD4^+^ T cells in DLN.

### Antibody responses

The levels of WNV-specific IgM and IgG were determined using an ELISA against purified WNV E protein [52]. Focus reduction neutralization assays were performed on Vero cells after mixing serial dilutions of serum with a fixed amount (100 FFU) of WNV [49].

### ELISPOT assay

WNV antigen-specific antibody secreting cells were quantified via an enzyme-linked immunospot assay [28]. Briefly, mixed cellulose esters filter plates (Millipore) were coated with 20 μg/ml of purified WNV E protein. Bone marrow cells were harvested from infected mice 90 days post infection, and 200,000 cells were seeded per well with 8 wells per mouse. Antibody spots were developed with chromogen substrate (3-amino-9-ethyl-carbazole) and wells were imaged and enumerated with an ImmunoSpot plate reader from Cellular Technology Ltd.

### Cell purification

To isolate naïve CD4^+^ T cells, splenocytes were isolated from mice and erythrocytes depleted with ACK lysis buffer (Invitrogen). Cells were then stained in buffer (PBS, 1% FBS, and 2 mM EDTA) with biotinylated antibodies against CD8, CD45R (B220), NK1.1, and MHC class II as well as fluorescently conjugated antibodies against CD4, CD44, and CD62L. Cells were washed with 10 mL of staining buffer and stained with anti-biotin magnetic beads (Miltenyi Biotec). Magnetically labeled cells were then depleted by passing through a LS magnetic column (Miltenyi Biotec) leaving roughly 90% pure CD4^+^ T cells. CD4^+^ CD44^-^ CD62L^+^ were then isolated on an Aria-II (BD Biosciences) fluorescence-activated cell sorter (see **S2 Fig**). B cells were isolated using a B Cell Isolation Kit (Miltenyi Biotech). Briefly, cells were stained with a biotinylated-antibody cocktail followed by anti-biotin labeled magnetic beads. Cells were loaded onto a magnetic LS column and the flow-through was collected.

### Adoptive cell transfers

Naïve CD4^+^ T cells or B cells from uninfected adult or old mice were sorted as detailed above. For cell tracking, isolated cells from adult and old donors were labeled differentially with vital dyes (CellTracker Orange CMTMR, CellTrace CFSE, or CellTrace Violet) according to the manufacturer recommendations. Importantly, dyes were switched between old and adult cells in some experiments and dye-dependent effects on cell trafficking were not observed under our staining conditions (data not shown). Isolated cells were incubated at 37°C and 5% CO_2_ in pre-warmed CO_2_-Independent Medium (Invitrogen) with 2 μM of CellTrace CFSE, 5 μM CellTracker Orange, or 10 μM of CellTrace Violet (Invitrogen) for 15 minutes. Subsequently, cells were centrifuged (300 x g, 5 minutes) and the pellet was resuspended in pre-warmed medium for another 30 minutes. The incubation was stopped after two washes with cold PBS. Finally, cells were counted and adjusted to equal number for homing or microscopy imaging experiments. Cells were transferred adoptively via an intravenous route into recipient mice that had been infected subcutaneously with WNV-KUNV 48 hours earlier. For transfer into TCR β/δ^-/-^ mice, 3 x 10^6^ sorted naïve CD4^+^ T cells were injected intravenously into each recipient mouse that had been infected subcutaneously with WNV-NY 48 hours earlier.

### T cell homing experiments

For analysis of T cell homing to the LN and spleen, naïve CD4^+^ T cells or B cells were sorted from adult and old mice and labeled with different dyes. Cells were suspended in PBS at a ratio 1:1 (adult to old) and injected intravenously (2 x 10^6^ from each donor). One hour post transfer, LN and spleens were harvested and processed into single cell suspensions. Cells were analyzed by flow cytometry on an LSRII (BD Biosciences).

### Intravital two-photon microscopy

We analyzed the trafficking behavior of lymphocytes in lymphoid organs using published methods for *ex vivo* (LN explants) and with minor modifications for *in vivo* imaging of cervical LNs [53]. Recipient mice (adult or old) infected with WNV-KUN 48 hours earlier were given 5 x 10^6^ cells CellTrace CFSE or Violet labeled naïve T cells (from adult and old donors). For *ex vivo* analysis, LN were harvested 6 to 8 hours later, glued to plastic cover slips with VetBond (3M), placed under the flow of warm (35°C to 37°C) oxygenated medium (DMEM, without phenol red) [54]. Intravital imaging of T cell extravasation was performed as described [55] with the following modifications. Mice were anesthetized with avertin (250 mg/kg) and connected to a MiniVet Mouse Ventilator (Harvard Apparatus) to reduce movement artifacts and improve mouse viability. The skin of the neck was opened and the cervical DLN exposed on a skin flap and the inner surface was attached to a plastic cover slip using VetBond with the LN protruding through a hole in the center. Imaging was performed on A custom-build two-photon microscope equipped with an Olympus 1.0 NA 20x water-immersion objective, a Vision II Coherent Ti:Sapphire laser and running ImageWarp (A&B Software) for hardware and acquisition control [56]. Dyes were excited at 820 nm and detected using 480 nm and 570 nm dichroic filters. T cell rolling in vessels was imaged at 25 f/sec and 3D images collected ~ 2 per minute with image dimensions of ~250 x 225 x 75 μm (X,Y,Z; 2.5 μm Z steps). Blood vessels in the LN were labeled with QTracker 655 (10 μl in 50 μl of PBS given intravenously, Life Technologies).

### Image analysis

Multidimensional data rendering and cell tracking were performed using Imaris software (version 7.3, Bitplane). For quantitative and statistical analysis of T cell motility we used either, T Cell Analyzer Software (version 1.7.0, Dr. J. Dempster, University of Strathclyde, UK) and an open-source software suite developed by Dr. J Textor (University of Ultrecht, The Netherlands). For quantitative and statistical analysis of T and B cell motility, we used the X and Y dimensions of the cell tracks to avoid bias arising from the intrinsically poorer Z resolution [57]. Displacement factors were defined as the distance between the endpoints of a track divided by the square root of its duration. The unpaired Mann-Whitney U test was used to compare track motility parameters. To control for variation between experiments, data were normalized prior to comparison by equalizing the medians across independent experiments. Hotelling's test for directionality was performed as described previously [58]. Briefly, cell tracks were broken down into steps (each step being a pair of consecutive cell positions), and the mean speed vector was computed per population of interest. Under the null hypothesis, the cells perform a random walk and the mean speed vector is zero in each dimension. Ellipses in **Figs 4K, 5E, 7F and 7J** show 95% confidence regions for the mean speed vector. If an ellipse does not contain the origin (0,0), then the null hypothesis is rejected for that population, and there is some directionality in the cell motion. For all cell populations studied in this paper, directionality was of a small magnitude (<1 μm/min at mean speeds of 5–10 μm/min).

### LN cell subset analysis

Adult or old draining popliteal LN were harvested from mice at the indicated times post infection. To generate a single cell suspension, LNs were placed in staining buffer and gently crushed with the back of a syringe plunger and cells passed through a 70 μm cell strainer. The total number of live cells was quantified by trypan blue exclusion in a hemocytometer. Cells were stained in buffer (PBS, 1% FBS, and 2 mM EDTA) with fluorescently conjugated antibodies for 30 minutes on ice. Cells were washed in buffer and then processed on an LSRII flow cytometer (BD Biosciences).

### Histology

Draining lymph nodes were harvested at 6 days post infection with WNV-KUN. DLNs were frozen in O.C.T. compound, and 9-μm-thick sections were cut with a cryostat. Sections were mounted on microscope slides and stained with anti–GL7-PE (1:50), anti–IgD-FITC (1:50), and DAPI (1 μg/ml) in PBS for 1 hour at room temperature. Slides were washed in PBS and a coverslip mounted on top of the stained tissue sections.

### Phenotyping of T cells

Splenocytes were isolated and erythrocytes were lysed with ACK buffer. Cells were stained with anti-CCR7 in staining buffer for 30 minutes at 37°C. Cells were washed and then incubated with anti-CD4, CD19, CD44, CD62L, LFA1, VLA4, PSGL1, and CXCR4 in staining buffer for 30 minutes on ice. Cells were processed on an LSRII flow cytometer. For filamentous actin staining, isolated CD4^+^ T cells were incubated with 100 ng/mL of CCL19 and CCL21 at 37°C in PBS + 0.1% BSA. Reactions were stopped by adding paraformaldehyde (3.6% final concentration), and cells were stained with anti-CD4, CD44, CD62L, and Alexa Fluor 647 phalloidin (Life Technologies). Cells were analyzed using an LSRII flow cytometer.

### Cytokine analysis

Mice were infected subcutaneously with 10^2^ PFU of WNV in the footpad. Draining popliteal LNs were collected at 24 or 48 hours post infection and placed in 200 μl of PBS containing 0.5% (w/v) BSA. DLN were homogenized and cytokines were quantified using a Bio-Plex Pro 23-plex group I cytokine kit (Bio-Rad) and Bio-Plex 200 (Bio-Rad). CXCL13 and CCL21 were measured using ELISA kits from R&D Systems.

### ICAM-1 binding and transwell migration assays

ICAM-1 Fc (R&D Systems) was adsorbed to a polystyrene Petri dish at 4 or 40 μg/ml in PBS. After a one-hour incubation at 37°C, plates were rinsed with PBS and non-specific binding sites were blocked with 1% human serum albumin (Sigma-Aldrich) for one hour at room temperature. Freshly isolated naïve CD4^+^ T cells (5.5 x 10^5^) were added to Petri dishes in 1.5 ml of L15 media containing 0.5% FBS and 10mM HEPES and supplemented with 200 ng/ml of CCL19 and CCL21. Dishes were incubated for one hour at 37°C and then fixed with 1% paraformaldehyde. Unbound cells were removed after extensive rinsing with PBS supplemented with 0.2% human serum albumin, 1 mM CaCl_2_, and 1 mM MgCl_2_. Dishes were imaged at 2.5X magnification and the number of adherent cells was counted. For the transwell migration assay, a 96-well modified Boyden chamber with 5 μm pore size was purchased (Neuro Probe). The top membrane was coated with 0, 4, or 40 μg/ml of ICAM-1 Fc in PBS for one hour at room temperature. CCL19 or CCL21 (0.1, 1, or 10 ng/ml) in migration buffer (RPMI 1640, 0.5% FBS, and 100U/ml penicillin-streptomycin) was plated in the bottom chamber. Freshly isolated naïve CD4^+^ T cells (5 x 10^4^) were added to the top chamber and incubated for 4 hours at 37°C. Subsequently, the membrane was removed and cells in bottom chamber were counted with a hemocytometer.

### Data analysis

All data was analyzed using Prism software (Graph Pad 6, San Diego, CA). Flow cytometry data was analyzed using FlowJo software (Tree Star Inc.). Kaplan-Meier survival curves were analyzed by the log rank test. All other statistical analyses were performed with Mann-Whitney or unpaired t-tests as indicated in the Figure legends.

## Supporting Information

S1 Fig Expression of co-stimulatory and activation markers on CD4^+^ T cells and T_FH_ cells from adult and old mice. CD4^+^ T cells (**A and C**) and T_FH_ cells (CD4^+^ PD1^+^ CXCR5^+^) (**B, D, and E**) from the DLN of mice infected for 6 days with WNV were analyzed by flow cytometry for the T cell co-stimulatory molecules CD28 (**A-B**) and OX40 (CD134) (**C-D**). The geometric mean fluorescent intensity was measured in individual experiments and normalized to the levels of cells from adult mice. T_FH_ cells were analyzed for levels of the chemokine receptor CXCR5 (**E**). Plotted is the mean plus the SEM with asterisks indicating statistical significance as judged by an unpaired t test (n.s., not-significant; **, *P* < 0.01; ***, *P* < 0.001). The results of **A-D** are pooled from two independent experiments with a total of 5 mice per group. The results of **E** are pooled from four independent experiments with a total of 11 mice per group. Included are representative flow cytometry and contour plots comparing expression of indicated surface antigens on cells from old and adult mice.
(TIF)

S2 Fig Flow cytometric sorting of naïve CD4^+^ T cells from adult and old mice. Naïve CD4^+^ T cells were identified as expressing low levels of CD44 and high levels of CD62L. The boxed area indicates cells that were sorted. Note the fraction of naïve CD4^+^ T cells is lower in old mice. One representative example of many is shown.
(TIF)

S3 Fig Decreased accumulation of CD4^+^ T cells and CD19^+^ B cells in DLN of old mice after infection with WNV-KUN. **A-C**. Adult and old mice were infected subcutaneously in the footpad with 10^3^ PFU of WNV-KUN. At day 2 after infection, the draining popliteal LN was harvested, and total cells were counted (**A**). Cells were stained with antibodies to detect specific lymphocyte populations including CD19^+^ B cells (**B**) and CD4^+^ T cells (**C**). The results are pooled from a total of 3 mice per group from a single experiment and data is expressed as the mean ± SD. Asterisks indicate statistical significance as judged by an unpaired t test (**, *P* < 0.01; ***, *P* < 0.001).
(TIF)

S4 Fig Additional analysis of defects in migration of naïve CD4^+^ T cells from old mice into inflamed LN. Analysis of movement parameters of adult and old donor naïve CD4^+^ T cells in explanted LN 6 to 8 hours post-transfer to recipient mice that had been infected with WNV-KUN 48 hours earlier. Individual differentially labeled adult and old naïve CD4^+^ T cells were observed and (**A**) mean track length, (**B**), track displacement length, (**C**) track straightness, (**D**) turning angle, and (**E**) slope were measured. The data are shown as a scatter plot and reflects three independent experiments. Asterisks indicate statistical significance as judged by the Mann-Whitney test (*, *P* < 0.05; **, *P* < 0.01; ***, *P* < 0.001, ****, *P* < 0.0001). The track straightness was calculated by dividing the distance a cell traveled from its starting point by the track length. Values of > 0.8 are commonly associated with chemotaxis, whereas values of < 0.5 are consistent with random cell migration. The slope indicates how fast the mean square displacement increases with time, and thus is a measure of maintenance of motility. **F-G**. Time-lapse image sequences of transferred adult and old naïve CD4^+^ T cells in explanted LN in recipient mice 48 h after WNV-KUN infection. Differentially labeled (blue = old, green = adult) naïve CD4+ T cells were adoptively transferred into WNV-KUNV infected adult recipient mice (48 h after infection) and DLN were harvested 6 to 8 h later followed by ex vivo imaging. Panel **F** presents all tracked adult and old cells during the first 12 minutes. Panel **G** and others presents time-lapse image sequences of adult and old cell tracking during first 12 minutes. Yellow and red arrows indicate which old and adult cells were analyzed and their starting points, respectively. Scale bar is indicated in white.
(TIF)

S5 Fig Surface staining of naïve CD4^+^ T cells from adult and old mice for adhesion molecules and chemokine receptors. Splenocytes from adult or old C57BL/6 mice were stained on their surface for markers for naïve CD4^+^ T cells (CD4^+^, CD44^-^, CD62L^+^) and for levels of adhesion molecules (**A**) (VLA-4 (CD49d), (**B**) L-selectin (CD62L), (**C**) LFA1 (CD11a), and (**D**) PSGL1 (CD162) or chemokine receptors ((**E**) CCR7 (CD197) or (**F**) CXCR4). The geometric mean fluorescent intensity was measured in individual experiments and normalized to the levels seen with cells from adult mice. The data is the average of at least two independent experiments comprising a total of 4 to 6 mice per group. None of the differences were statistically significant. Also shown are representative histograms of each surface marker on naïve CD4^+^ T cells from an adult and old mouse along with the fluorescence minus one (FMO) control. Because CD44- CD62L^+^ cells were gated for the flow cytometric analysis, an FMO control is not included in the L-selectin (CD62L) histogram.
(TIF)

S6 Fig Cytokine-induced cytoskeletal rearrangement and ICAM-1 binding of naïve CD4^+^ T cells from adult and old mice. **A**. CD4^+^ T cells were isolated from adult and old mice and stimulated with 100 ng/mL of both CCL19 and CCL21. Cells were fixed at the indicated time post stimulation and stained for naïve CD4^+^ T cell markers (CD4^+^ CD44^-^ CD62L^+^) and phalloidin-A647 (which binds to filamentous actin). Cells were analyzed by flow cytometry and the geometric mean fluorescent intensity was measured. Shown is the mean + SEM from three independent experiments. **B-C**. Naïve CD4^+^ T cells were isolated from adult or old mice. ICAM-1 Fc (0, 4, or 40 μg/ml) was coated on (**B**) polystyrene Petri dishes or (**C**) 5 μm transwell membranes. **B.** Cells were added to the ICAM-1 coated Petri dishes in the presence of 200 ng/ml of CCL19 and CCL21. After a one-hour incubation, adherent cells were counted. Plotted is the number of adherent cells (mean ± SEM) minus the background (to 0 μg/ml of ICAM-1 Fc). Data is pooled from three independent experiments. **C.** CCL19 and CCL21 (0.1, 1, or 10 ng/ml) were plated in the bottom compartment of a Boyden chamber and cells were added to the top chamber above the transwell membrane insert. The number of cells migrating through the membrane was quantified and normalized to the number observed at 40 μg/mL ICAM-1 Fc in the presence of 1 ng/mL of CCL19 and CCL21. Plotted is the mean number of migrating cells (± SEM) from three independent experiments.
(TIF)

S1 Movie Intravital microscopy showing differential movement of adoptively transferred naïve CD4^+^ T cells from adult and old mice across the HEV. Naïve CD4^+^ T cells from adult and old mice were sorted, differentially labeled with fluorescent dyes (adult = green; old = blue) and adoptively transferred into adult mice that had been infected subcutaneously with WNV-KUN 48 hours earlier. One hour later, the DLN was evaluated by intravital microscopy and vascular structures were visualized with labeled quantum dots. One representative experiment of three is shown. The time of analysis and scale bar are indicated.
(MOV)

S2 Movie Higher-magnification images showing differential movement of adoptively transferred naïve CD4^+^ T cells from adult and old mice across the HEV. These images was obtained from **S1 Movie** at higher magnification to highlight differential trafficking capacity of naïve CD4^+^ T cells from adult and old mice. The time of analysis and scale bar are indicated.
(MOV)

S3 Movie Intravital microscopy showing differential movement of adoptively transferred naïve CD4^+^ T cells from adult and old mice in explanted LN. Naïve CD4^+^ T cells from adult and old mice were sorted, differentially labeled with fluorescent dyes (adult = green; old = blue) and adoptively transferred into adult mice that had been infected subcutaneously with WNV-KUN 48 hours earlier. Six hours later, the DLN was explanted and evaluated by intravital microscopy. Measurements of velocity and distance movement by individual naïve CD4^+^ T cells (see **Fig 4**and **S4 Fig**) were obtained from these videos. One representative experiment of several independent trials is shown. The time of analysis and scale bar are indicated.
(MOV)

S4 Movie Intravital microscopy showing similar movement patterns of adoptively transferred CD19^+^ B cells from adult and old mice in explanted LN. CD19^+^ B cells from adult and old mice were sorted, differentially labeled with fluorescent dyes (adult = green; old = blue) and adoptively transferred into adult mice that had been infected subcutaneously with WNV-KUN 48 hours earlier. Six hours later, the DLN was explanted and evaluated by intravital microscopy. Measurements of velocity and distance movement by individual CD19^+^ B cells (see **Fig 7**) were obtained from these videos. One representative experiment of several independent trials is shown. The time of analysis and scale bar are indicated.
(MOV)
