# Supplementary figures and images for: Age-Dependent Cell Trafficking Defects in Draining Lymph Nodes Impair Adaptive Immunity and Control of West Nile Virus Infection
Source: PLoS Pathog. 2015 Jul 23;11(7):e1005027. doi: 10.1371/journal.ppat.1005027 (PMC4512688; doi:10.1371/journal.ppat.1005027)

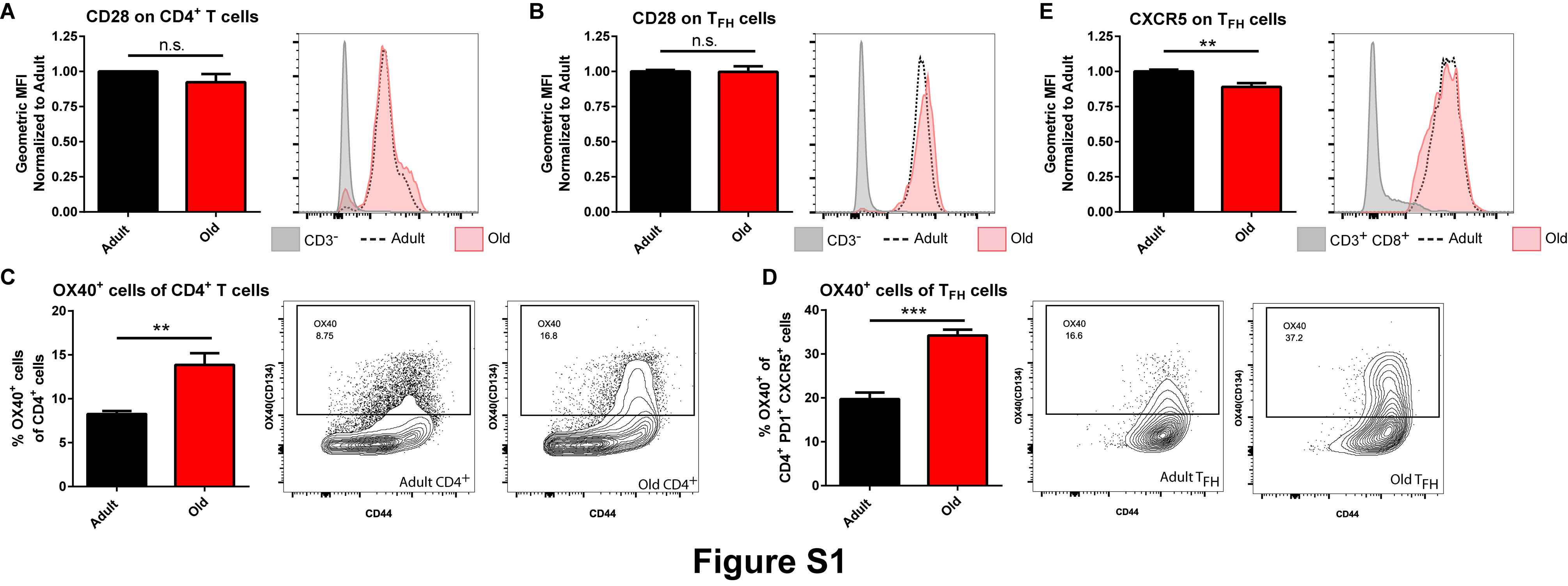

Supplement: S1 Fig — CD4+ T cells (A and C) and TFH cells (CD4+ PD1+ CXCR5+) (B, D, and E) from the DLN of mice infected for 6 days with WNV were analyzed by flow cytometry for the T cell co-stimulatory molecules CD28 (A-B) and OX40 (CD134) (C-D). The geometric mean fluorescent intensity was measured in individual experiments and normalized to the levels of cells from adult mice. TFH cells were analyzed for levels of the chemokine receptor CXCR5 (E). Plotted is the mean plus the SEM with asterisks indicating statistical significance as judged by an unpaired t test (n.s., not-significant; **, P < 0.01; ***, P < 0.001). The results of A-D are pooled from two independent experiments with a total of 5 mice per group. The results of E are pooled from four independent experiments with a total of 11 mice per group. Included are representative flow cytometry and contour plots comparing expression of indicated surface antigens on cells from old and adult mice. (TIF) [file ppat.1005027.s001.tif]

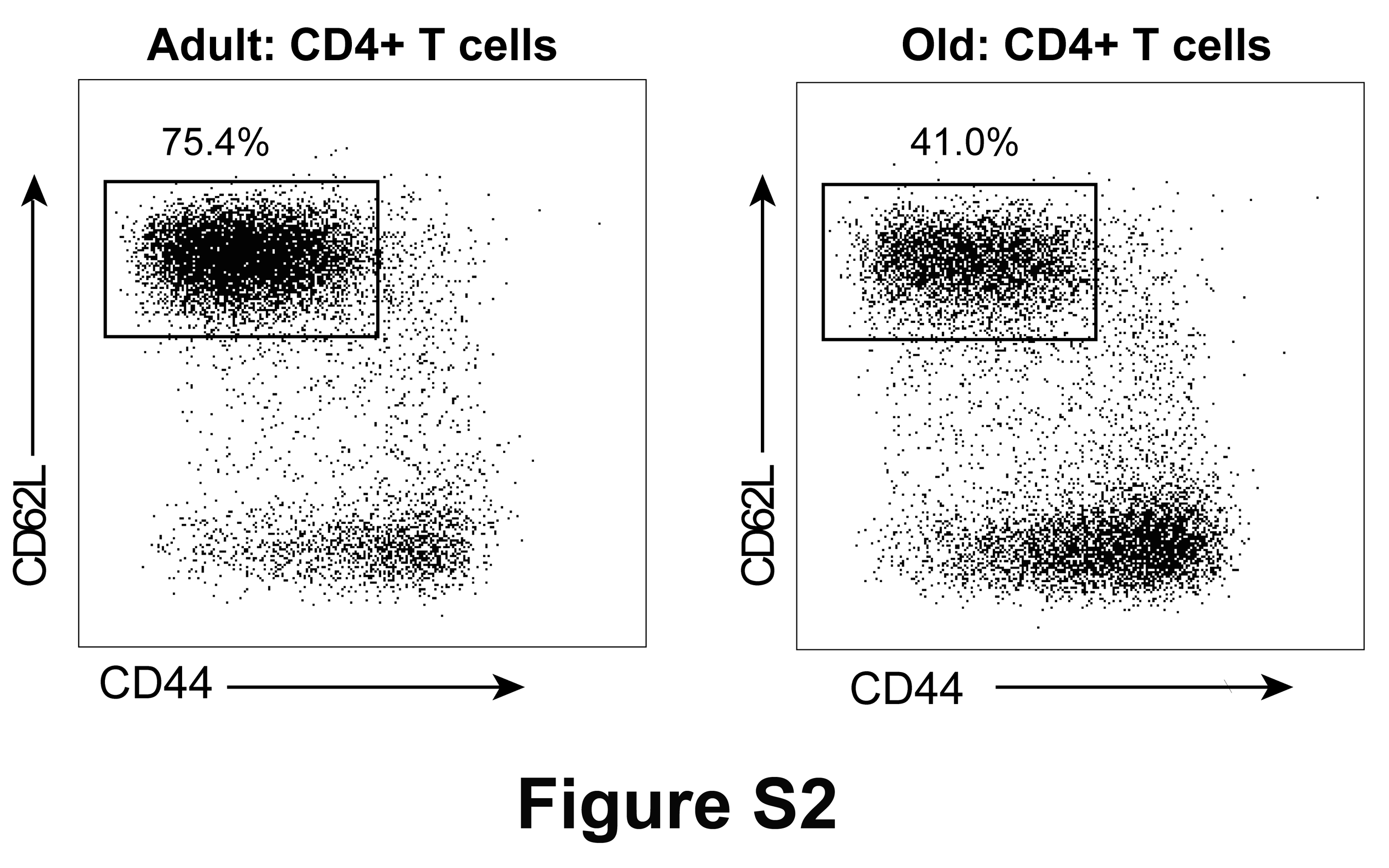

Supplement: S2 Fig — Naïve CD4+ T cells were identified as expressing low levels of CD44 and high levels of CD62L. The boxed area indicates cells that were sorted. Note the fraction of naïve CD4+ T cells is lower in old mice. One representative example of many is shown. (TIF) [file ppat.1005027.s002.tif]

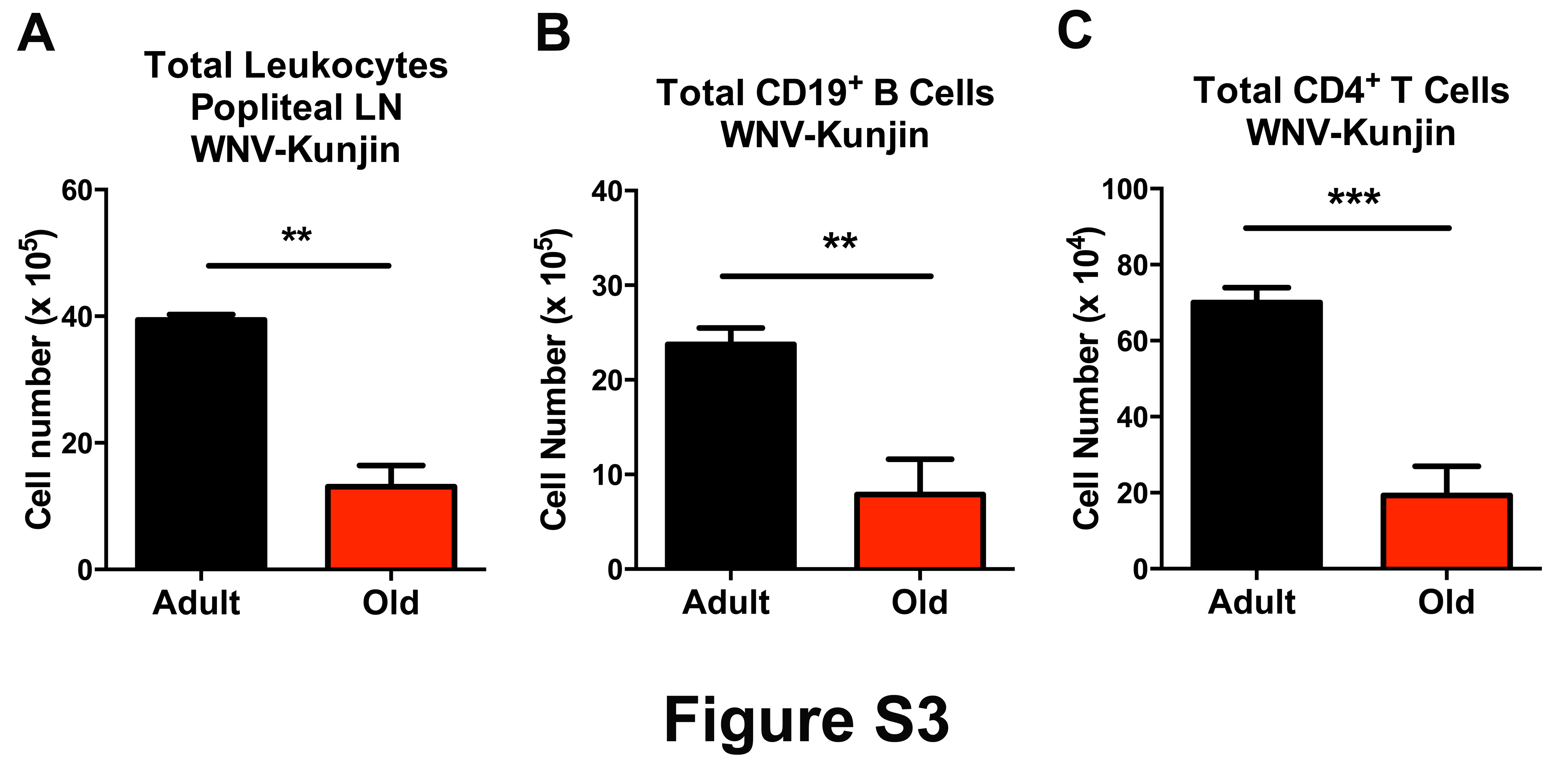

Supplement: S3 Fig — A-C. Adult and old mice were infected subcutaneously in the footpad with 103 PFU of WNV-KUN. At day 2 after infection, the draining popliteal LN was harvested, and total cells were counted (A). Cells were stained with antibodies to detect specific lymphocyte populations including CD19+ B cells (B) and CD4+ T cells (C). The results are pooled from a total of 3 mice per group from a single experiment and data is expressed as the mean ± SD. Asterisks indicate statistical significance as judged by an unpaired t test (**, P < 0.01; ***, P < 0.001). (TIF) [file ppat.1005027.s003.tif]

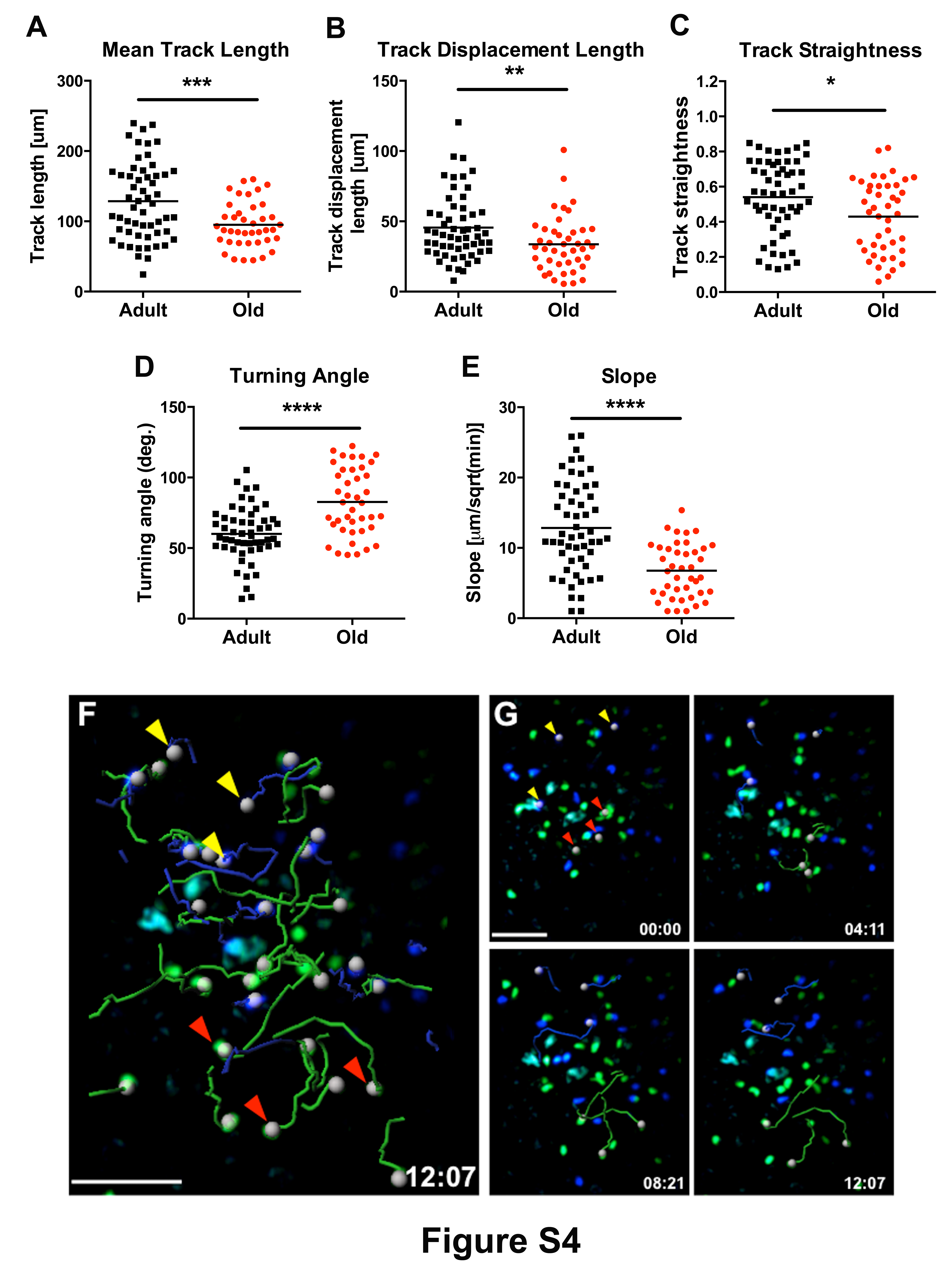

Supplement: S4 Fig — Analysis of movement parameters of adult and old donor naïve CD4+ T cells in explanted LN 6 to 8 hours post-transfer to recipient mice that had been infected with WNV-KUN 48 hours earlier. Individual differentially labeled adult and old naïve CD4+ T cells were observed and (A) mean track length, (B), track displacement length, (C) track straightness, (D) turning angle, and (E) slope were measured. The data are shown as a scatter plot and reflects three independent experiments. Asterisks indicate statistical significance as judged by the Mann-Whitney test (*, P < 0.05; **, P < 0.01; ***, P < 0.001, ****, P < 0.0001). The track straightness was calculated by dividing the distance a cell traveled from its starting point by the track length. Values of > 0.8 are commonly associated with chemotaxis, whereas values of < 0.5 are consistent with random cell migration. The slope indicates how fast the mean square displacement increases with time, and thus is a measure of maintenance of motility. F-G. Time-lapse image sequences of transferred adult and old naïve CD4+ T cells in explanted LN in recipient mice 48 h after WNV-KUN infection. Differentially labeled (blue = old, green = adult) naïve CD4+ T cells were adoptively transferred into WNV-KUNV infected adult recipient mice (48 h after infection) and DLN were harvested 6 to 8 h later followed by ex vivo imaging. Panel F presents all tracked adult and old cells during the first 12 minutes. Panel G and others presents time-lapse image sequences of adult and old cell tracking during first 12 minutes. Yellow and red arrows indicate which old and adult cells were analyzed and their starting points, respectively. Scale bar is indicated in white. (TIF) [file ppat.1005027.s004.tif]

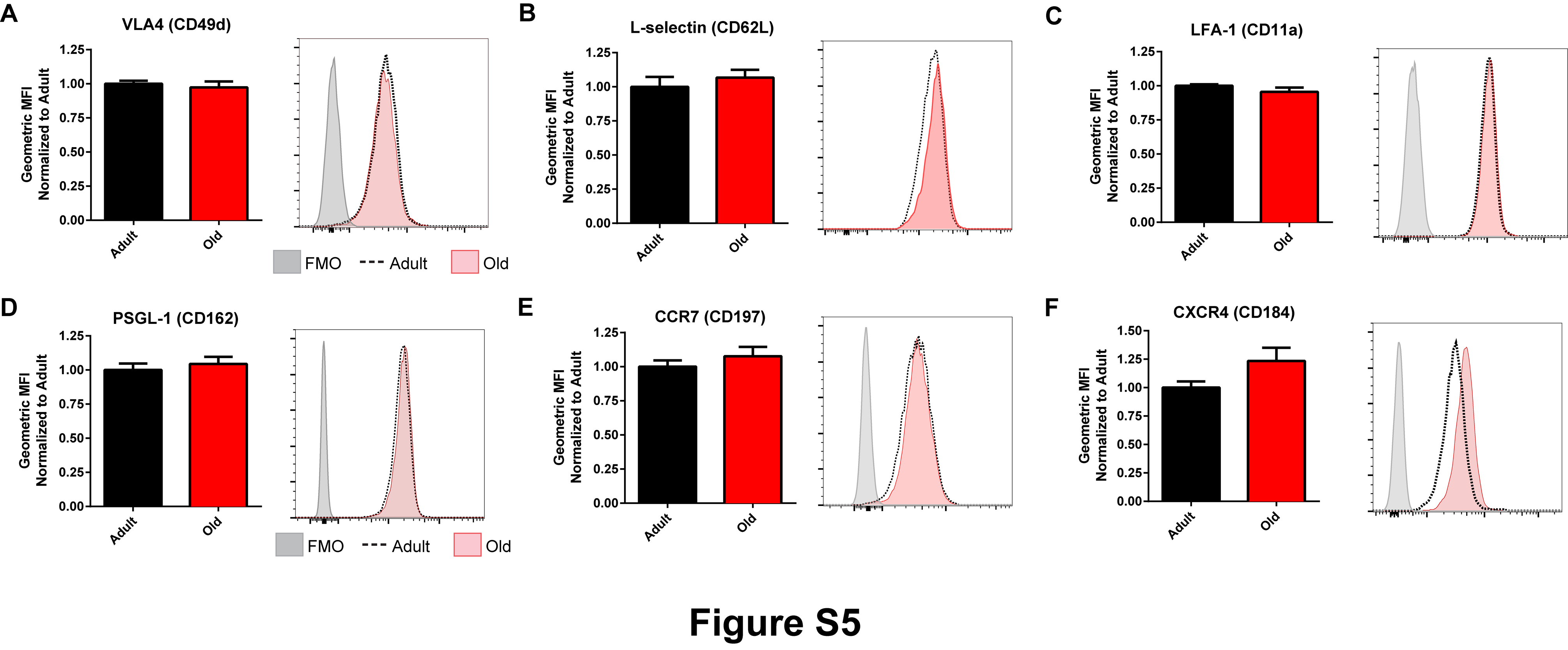

Supplement: S5 Fig — Splenocytes from adult or old C57BL/6 mice were stained on their surface for markers for naïve CD4+ T cells (CD4+, CD44-, CD62L+) and for levels of adhesion molecules (A) (VLA-4 (CD49d), (B) L-selectin (CD62L), (C) LFA1 (CD11a), and (D) PSGL1 (CD162) or chemokine receptors ((E) CCR7 (CD197) or (F) CXCR4). The geometric mean fluorescent intensity was measured in individual experiments and normalized to the levels seen with cells from adult mice. The data is the average of at least two independent experiments comprising a total of 4 to 6 mice per group. None of the differences were statistically significant. Also shown are representative histograms of each surface marker on naïve CD4+ T cells from an adult and old mouse along with the fluorescence minus one (FMO) control. Because CD44- CD62L+ cells were gated for the flow cytometric analysis, an FMO control is not included in the L-selectin (CD62L) histogram. (TIF) [file ppat.1005027.s005.tif]

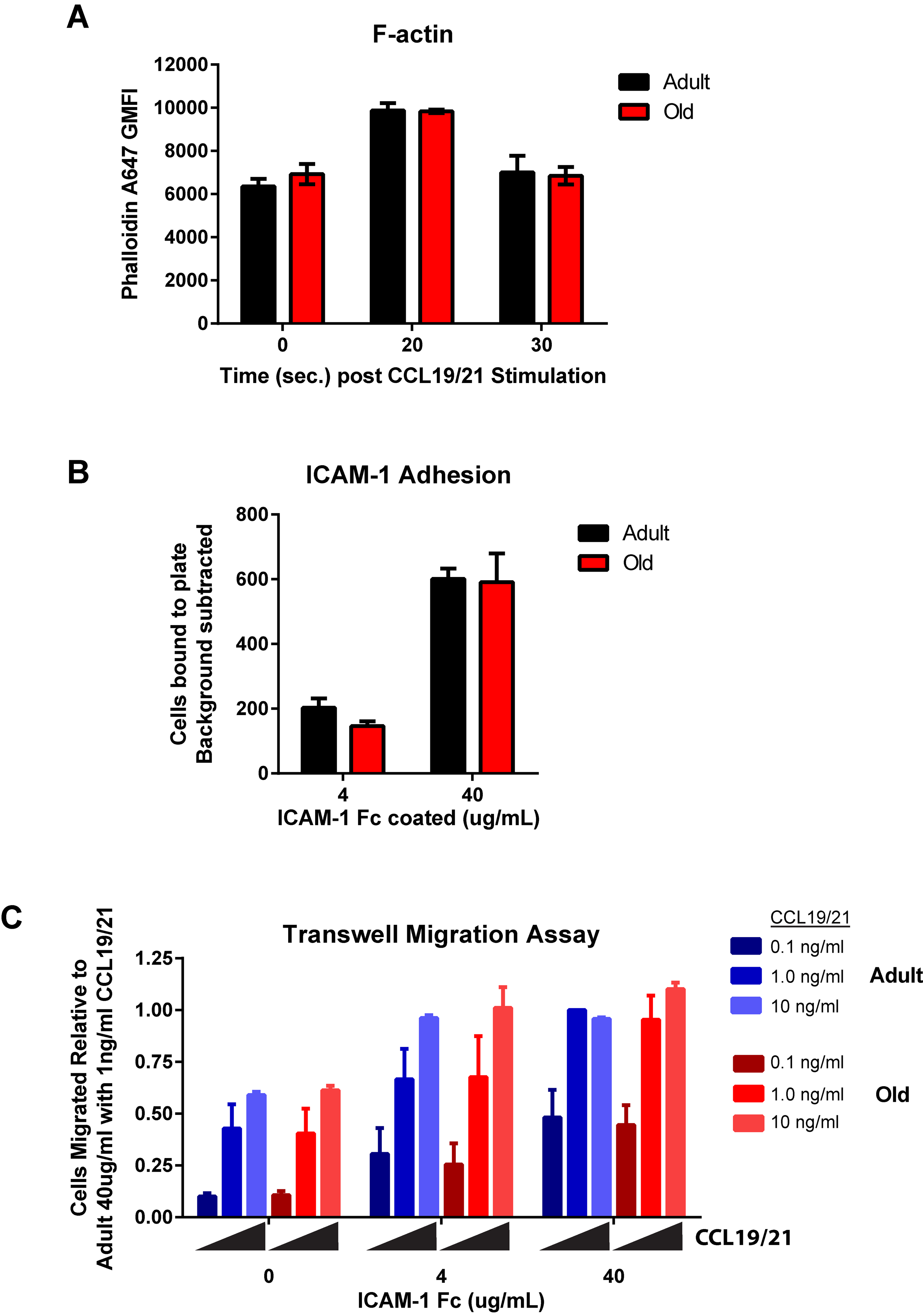

Supplement: S6 Fig — A. CD4+ T cells were isolated from adult and old mice and stimulated with 100 ng/mL of both CCL19 and CCL21. Cells were fixed at the indicated time post stimulation and stained for naïve CD4+ T cell markers (CD4+ CD44- CD62L+) and phalloidin-A647 (which binds to filamentous actin). Cells were analyzed by flow cytometry and the geometric mean fluorescent intensity was measured. Shown is the mean + SEM from three independent experiments. B-C. Naïve CD4+ T cells were isolated from adult or old mice. ICAM-1 Fc (0, 4, or 40 μg/ml) was coated on (B) polystyrene Petri dishes or (C) 5 μm transwell membranes. B. Cells were added to the ICAM-1 coated Petri dishes in the presence of 200 ng/ml of CCL19 and CCL21. After a one-hour incubation, adherent cells were counted. Plotted is the number of adherent cells (mean ± SEM) minus the background (to 0 μg/ml of ICAM-1 Fc). Data is pooled from three independent experiments. C. CCL19 and CCL21 (0.1, 1, or 10 ng/ml) were plated in the bottom compartment of a Boyden chamber and cells were added to the top chamber above the transwell membrane insert. The number of cells migrating through the membrane was quantified and normalized to the number observed at 40 μg/mL ICAM-1 Fc in the presence of 1 ng/mL of CCL19 and CCL21. Plotted is the mean number of migrating cells (± SEM) from three independent experiments. (TIF) [file ppat.1005027.s006.tif]
